# Molecular mechanism of antagonist recognition and regulation of the α_1A_-adrenoceptor

**DOI:** 10.1016/j.jbc.2025.110348

**Published:** 2025-06-06

**Authors:** Sisi Liu, Haizhan Jiao, Yuyong Tao, Dandan Wang, Qiong Guo

**Affiliations:** 1 Department of Laboratory Medicine, The First Affiliated Hospital of USTC, MOE Key Laboratory for Membraneless Organelles and Cellular Dynamics, Hefei National Center for Cross-disciplinary Sciences, Biomedical Sciences and Health Laboratory of Anhui Province, Center for Advanced Interdisciplinary Science and Biomedicine of IHM, Division of Life Sciences and Medicine, University of Science and Technology of China, Hefei, China; 2 Kobilka Institute of Innovative Drug Discovery, School of Medicine, The Chinese University of Hong Kong, Shenzhen, China

**Keywords:** G protein–coupled receptor, doxazosin, silodosin, antagonists, α_1A_-adrenoceptor, cryo-EM

## Abstract

The α_1_-adrenoceptor (α_1_AR) is a critically important class of G protein–coupled receptors, comprising 3 subtypes: α_1A_AR, α_1B_AR, and α_1D_AR. Currently, drugs targeting α_1_AR have been used in the treatment of various diseases. Notably, antagonists of α_1_AR play a pivotal role in the management of benign prostatic hyperplasia. In recent years, researchers have developed selective antagonists for the α_1A_AR subtype that have a minimal impact on blood pressure for the treatment of benign prostatic hyperplasia. However, these agents still exhibit certain side effects, necessitating the continuous development of new medications to mitigate adverse reactions while achieving more precise regulation. We report the cryo-EM structures of the α_1_AR-selective antagonist doxazosin and the α_1A_AR subtype–selective antagonist silodosin in complex with α_1A_AR, demonstrating that M292^6.55^ and V185^5.39^ are key residues that confer subtype selectivity to silodosin. In addition, modifications to α_1B_AR enhanced silodosin's inhibitory efficacy against α_1B_AR. These findings deepen our understanding of the recognition patterns of α_1A_AR antagonists, revealing the molecular principles underlying the selective binding of silodosin to α_1A_AR and promoting further research and development of subtype-selective drugs targeting α_1A_AR.

Adrenoceptors constitute a significant class of G protein–coupled receptors (GPCRs), primarily modulating diverse physiological processes through their interaction with epinephrine and norepinephrine. Adrenoceptors include α-adrenoceptors (α_1_ and α_2_) and β-adrenoceptors (β1, β2, and β3) (1, 2, 3). Among them, the α_1_-adrenoceptor (α_1_AR) comprises 3 subtypes: α_1A_AR, α_1B_AR, and α_1D_AR, which mediate signal transduction *via* the activation of Gq/11 proteins (1, 4, 5). Antagonists targeting α_1_AR have been primarily utilized clinically for management of hypertension and benign prostatic hyperplasia (BPH) (6, 7). In hypertension treatment, α_1_AR antagonists bind to vascular smooth muscle, facilitating vasodilation and thereby reducing blood pressure, but their therapeutic efficacy is generally inferior to other antihypertensive agents, rendering them unsuitable as first-line monotherapy (7, 8, 9, 10). In treating BPH, α_1_AR antagonists alleviate urinary obstruction symptoms by relaxing prostatic smooth muscle (6, 7). The nonselective α-adrenoceptor antagonist phenoxybenzamine, reported effective for BPH treatment in 1976, exhibited severe adverse reactions. Therefore, subsequent research focused on developing selective and long-acting α_1_AR antagonists (11, 12, 13). The first selective α_1_AR antagonist, prazosin, demonstrated good tolerability but lacked sufficient dosing convenience for widespread clinical use (11, 14). Later, long-acting selective α_1_AR antagonists approved by the Food and Drug Administration, such as alfuzosin, doxazosin, and terazosin, improved dosing convenience but suffered from limited subtype selectivity and were associated with hypotensive side effects (11, 15, 16, 17, 18). Tamsulosin, the first subtype-selective α_1A_AR antagonist, exhibited approximately 10-fold higher affinity for α_1A_AR in prostate tissue compared with α_1B_AR in vascular tissues, but its relatively modest selectivity still posed a risk for cardiovascular side effects (11, 19, 20). Silodosin, a next-generation antagonist with higher α_1A_AR subtype selectivity, demonstrated improved efficacy and safety profiles, although some side effects, such as orthostatic hypotension, persist (21, 22, 23). Future research should aim at improving therapeutic efficacy of α_1_AR antagonists while minimizing side effects.

In recent years, structure-based drug design has become a critical approach in drug discovery, enabling precise ligand design through detailed structural analysis of drug–receptor complexes (24, 25, 26). Structures of α_1A_AR bound to agonists, including noradrenaline, oxymetazoline, and A61603, have previously been reported; however, antagonist-bound structures remain limited, with only the high-affinity antagonist tamsulosin bound to α_1A_AR structurally characterized thus far (27, 28). Structural information on highly α_1A_AR-selective antagonists remains elusive. Therefore, elucidating the structural complexes of additional antagonists bound to α_1A_AR is crucial for comprehending the molecular principles underlying ligand antagonism and selectivity. In this study, we determined the structures of α_1A_AR in complex with the α_1_AR-selective antagonist doxazosin and the α_1A_AR subtype–selective antagonist silodosin. We explored the molecular mechanisms underpinning antagonist recognition and binding selectivity, especially highlighting structural determinants governing silodosin's subtype selectivity. Our findings provide molecular insights for future design and optimization of α_1A_AR subtype–selective therapeutic agents.

## Results

### Structural determination of α*_1A_AR bound to distinct antagonists*

**Figure 1:**
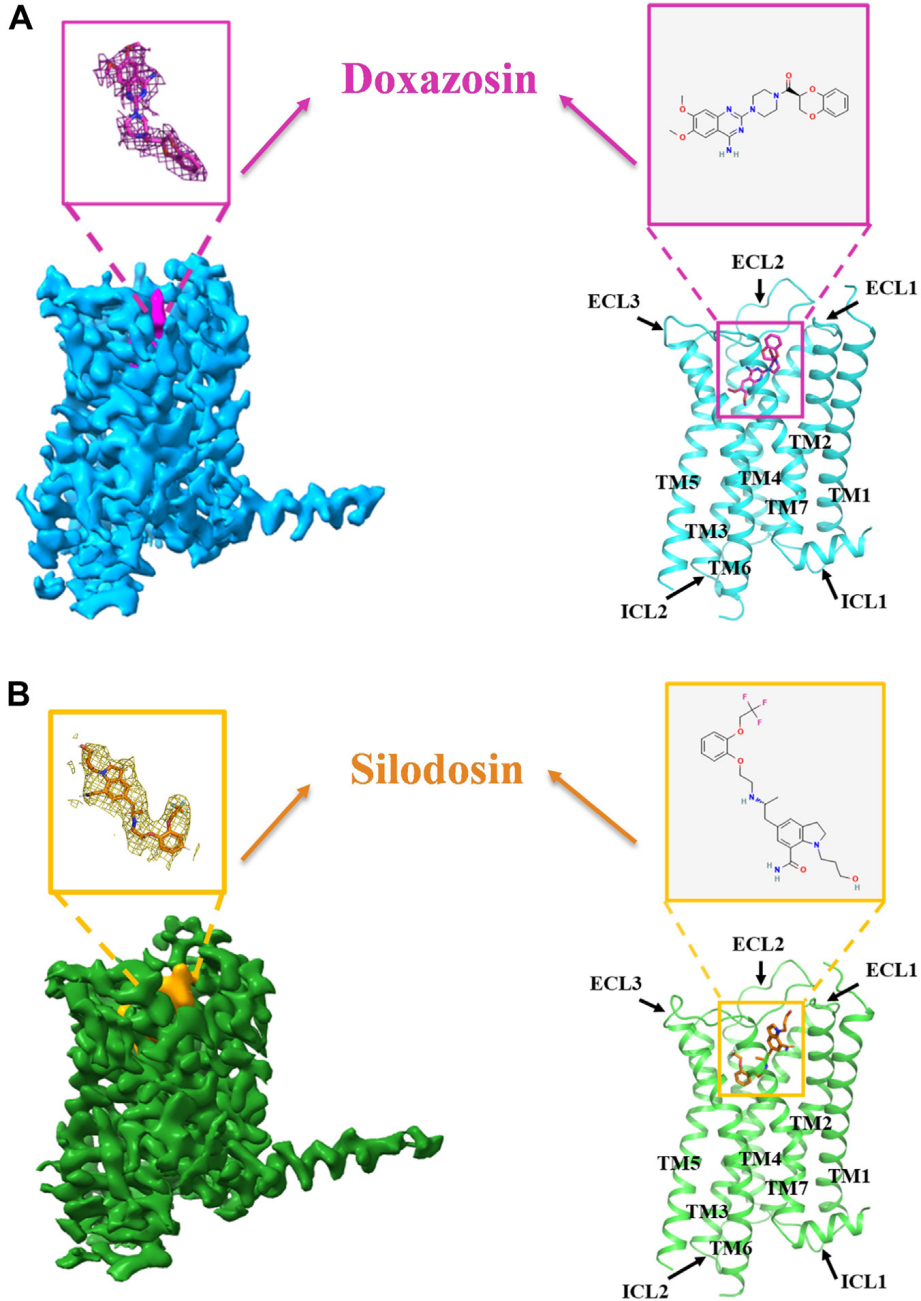
**Cryo-EM maps and overall structures of α_1A_AR in complex with doxazosin and silodosin**. *A*, the cryo-EM map (*left*) and overall structure after refinement in the map (*right*) of the α_1A_AR–doxazosin complex, with doxazosin represented in *pink* and α_1A_AR in *light blue*. The chemical structure of doxazosin was obtained from PubChem (https://pubchem.ncbi.nlm.nih.gov/compound/6604576#section=2D-Structure). *B*, the cryo-EM map (*left*) and overall structure after refinement in the map (*right*) of the α_1A_AR–silodosin complex, with silodosin depicted in *orange* and α_1A_AR in *green*. The chemical structure of silodosin was obtained from PubChem (https://pubchem.ncbi.nlm.nih.gov/compound/5312125#section=2D-Structure). *A* and *B*, unless otherwise stated, this color scheme will consistently be utilized in all subsequent figures. The mBRIL, Fab, and Glue protein are not shown.

To obtain the complex structures of α_1A_AR bound to the antagonists doxazosin and silodosin, we employed a universal GPCR structure determination method developed by our research group to assemble α_1A_AR-mBRIL–Fab–G7-13 complexes *in vitro* (29). We inserted mBRIL between TM5 and TM6 of α_1A_AR and introduced a K3-ALFA tag after helix 8 of the receptor. Furthermore, G7-13 was selected as the glue molecule for complex assembly (Fig. S1, *A*–*B*) (29). Subsequent 2D analysis of the samples revealed that both complexes exhibited favorable particle characteristics, with clear density observed in the transmembrane region (Fig. S1, *C*–*D*). Following extensive data collection and processing, we obtained cryo-EM density maps at resolutions of 2.99 Å and 3.19 Å. We used the structure of the α_1A_AR–mBRIL fusion protein predicted by AlphaFold2 as the initial template to build and refine structural models of the complexes, ultimately generating the final structural models of the doxazosin–α_1A_AR and silodosin–α_1A_AR complexes (Fig. 1, Figs. S2–S3 and Table S1). The complex structures indicate that both doxazosin and silodosin bind to the orthosteric binding site of α_1A_AR (Fig. 1). Moreover, the RMSD of Cα atoms between the 2 complex structures is 0.46 Å, suggesting that the overall conformation of α_1A_AR is nearly identical when bound to either antagonist.

### Structural rearrangements during α_1A_AR activation

**Figure 2:**
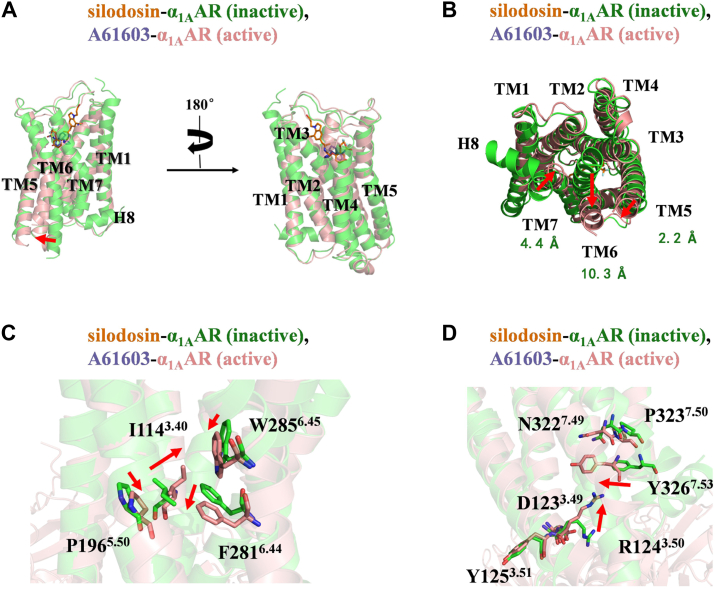
**Conformational changes during α_1A_AR activation compared with the inactive state of α_1A_AR bound to silodosin**. *A*, side-view comparison of α_1A_AR bound to silodosin and A61603 (*blue–purple*) (PDB ID: 8THK). The α_1A_AR bound to A61603 is depicted in *light pink*. The *red arrow* illustrates the outward displacement of TM6 upon binding with A61603. *B*, comparison of α_1A_AR bound to silodosin and A61603 on the cytoplasmic face. The displacement distances of TM6, TM7, and TM5 were measured using the Cα atoms of residues A270^6.33^, Y326^7.53^, and R213^5.67^, respectively. These are indicated in the figure with *green labels* and highlighted with *red arrows*. *C*, conformational changes of the toggle switch W285^6.48^ and PIF motif. Residue shifts are indicated by *red arrows*. W285^6.48^ (position of the seventh carbon on the indole ring) moves downward by 1.6 Å, P196^5.50^ (position of Cα atom) shifts downward by 1.4 Å, and F281^6.44^ (position of the fourth carbon on the benzene ring) moves outward by 4.9 Å. *D*, conformational changes of the NPxxY and DRY motifs. Residue shifts are marked by *red arrows*. R124^3.50^ (position of the central carbon of the guanidinium group) shifts upward by 6.3 Å, and Y326^7.53^ (position of Cα atom) moves inward toward the helix axis by 4.4 Å. PDB, Protein Data Bank.

To identify conformational changes associated with receptor activation, we compared the antagonist-bound α_1A_AR complexes (silodosin–α_1A_AR and doxazosin–α_1A_AR) with the agonist-bound complex (A61603–α_1A_AR) (28). Upon activation by A61603, the intracellular region of TM6 shifted outward by approximately 10 Å, whereas TM5 and TM7 moved inward by approximately 2 Å and 4 Å, respectively (Fig. 2, *A* and *B*, Figs. S4, *A*–*B* and Movie S1–S2). This is highly consistent with the characteristic features of GPCR activation (27, 28, 30, 31). Further structural analysis revealed a downward shift of approximately 2 Å in the toggle switch residue W285^6.48^ (superscripts indicate Ballesteros–Weinstein numbering (32)) upon agonist binding (Figs. 2*C*, S4*C* and Movie S1–S2). This movement was transmitted to the adjacent PIF motif, where residue P196^5.50^ shifted downward by roughly 1 Å, I114^3.40^ rotated inward toward the helical core, and F281^6.44^ rotated significantly outward by approximately 5 Å (Figs. 2*C*, S4*C* and Movie S1–S2). These rearrangements propagated toward the DRY motif, resulting in an upward displacement of approximately 6 Å in residue R124^3.49^ (Figs. 2*D*, S4*D* and Movie S1–S2). Furthermore, within the NPxxY motif of TM7, Y326^7.53^ exhibited a prominent inward shift of approximately 4 Å (Figs. 2*D*, S4*D* and Movie S1–S2). Collectively, these coordinated conformational shifts culminated in a substantial outward displacement of TM6, creating an intracellular cavity critical for accommodating and recruiting downstream signaling proteins.

### Recognition mechanism of doxazosin with α_1A_AR

**Figure 3:**
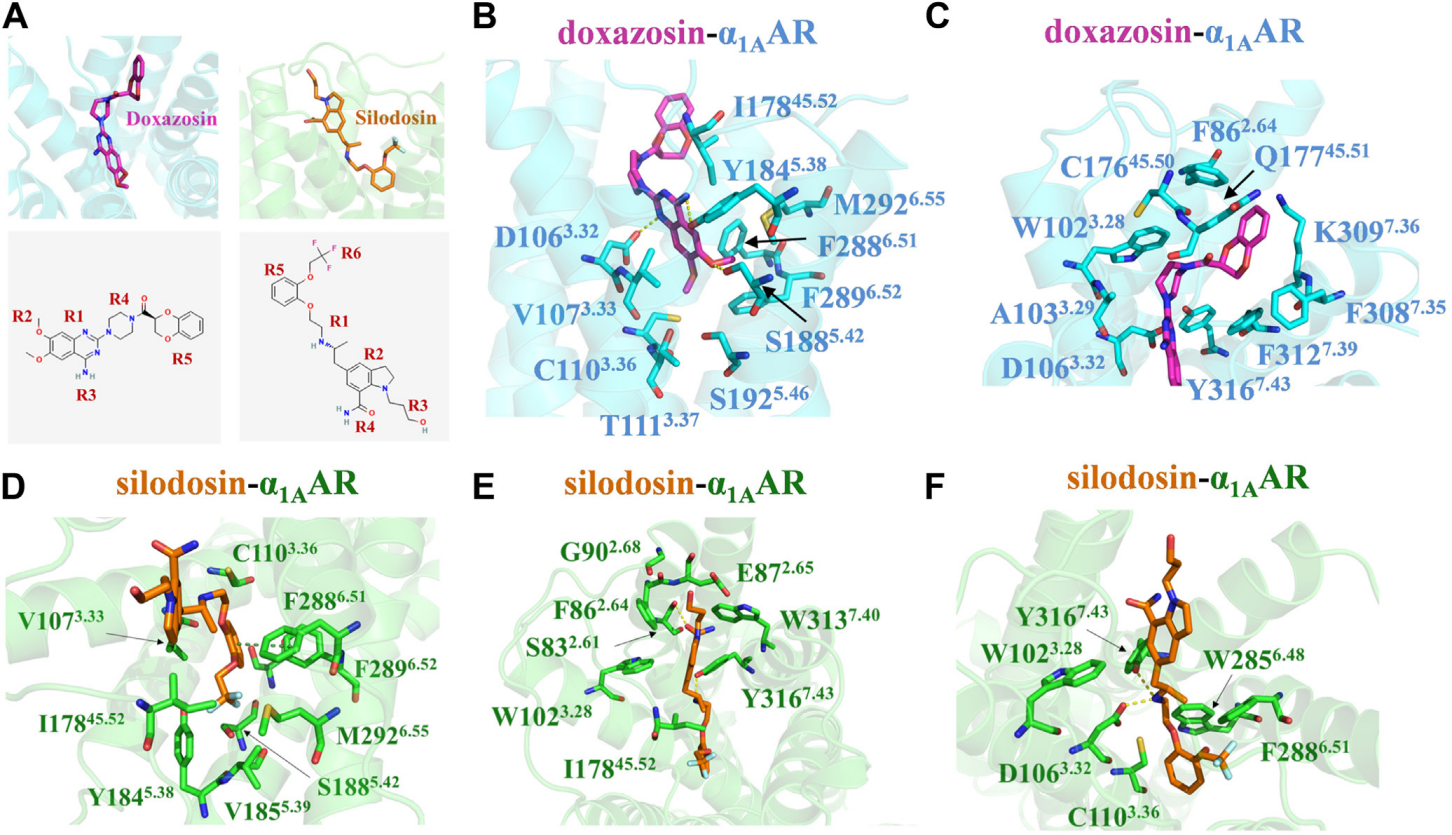
**Interactions of antagonists within the ligand-binding pocket of α_1A_AR**. *A*, the binding modes of doxazosin and silodosin in α_1A_AR as well as the chemical structures of doxazosin (https://pubchem.ncbi.nlm.nih.gov/compound/6604576#section=2D-Structure) and silodosin (https://pubchem.ncbi.nlm.nih.gov/compound/5312125#section=2D-Structure). *B*, details of the interactions between the dimethoxyquinazoline moiety of doxazosin and α_1A_AR. Doxazosin interacts with surrounding residues, forming hydrogen bonds with D106^3.32^, Y184^5.38^, and S188^5.42^. *C*, details of the interactions between the 1,4-benzodioxin-2-carbonyl and piperazine moieties of doxazosin and α_1A_AR. *D*, interactions between the phenoxy and trifluoroethoxy moieties of silodosin and α_1A_AR. The phenoxy group of silodosin forms a π–π interaction with F289^6.52^. *E*, details of the interactions between the indole–carboxamide and hydroxypropyl moieties of silodosin and α_1A_AR. The indolocarboxamide moiety of silodosin forms a hydrogen bond with S83^2.61^. *F*, details of the interactions between the main chain ethylaminopropyl moiety of silodosin and α_1A_AR. Silodosin forms polar interactions with D106^3.32^ and Y316^7.43^*via* its amine group. *B*–*F*, hydrogen bonds are represented as *yellow dashed lines*, whereas π–π interactions are depicted as *deep-green lines*.

Doxazosin is a selective α_1_AR antagonist that belongs to the quinazoline class of drugs. The quinazoline moiety (R1) consists of a benzene ring fused to a pyrimidine ring, substituted by dimethoxy (R2), amino (R3), and piperazine (R4) groups; the piperazine group is further substituted by a 1,4-benzodioxin-2-carbonyl substituent (R5) (Fig. 3*A*) (33, 34).

The structure of the doxazosin–α_1A_AR complex reveals that doxazosin adopts an inverted L-shaped conformation within the binding pocket of α_1A_AR, establishing extensive interactions with surrounding residues (Fig. 3, *A–C*). Specifically, the dimethoxyquinazoline moiety of doxazosin inserts into the orthosteric site of α_1A_AR, forming hydrophobic contacts with multiple residues, including C110^3.36^, V107^3.33^, T111^3.37^, F288^6.51^, F289^6.52^, I178^5.52^, M292^6.55^, and Y184^5.38^ (Fig. 3*B*). In addition to hydrophobic interactions, 3 key hydrogen bonds are observed: the oxygen atom of the dimethoxy group forms a hydrogen bond with S188^5.42^; the nitrogen atom in the pyrimidine ring forms a hydrogen bond with D106^3.32^; and the nitrogen atom in the amine group forms a hydrogen bond with Y184^5.38^ (Fig. 3*B*). Notably, D106^3.32^ is highly conserved and critical for ligand binding across aminergic receptors, playing a pivotal role in receptor affinity and function (28, 35). The other 2 moieties of doxazosin, the 1,4-benzodioxane-2-carbonyl and piperazine groups, are accommodated by α_1A_AR residues D106^3.32^, W102^3.28^, A103^3.29^, C176^45.50^, Q177^45.51^, F312^7.39^, K309^7.36^, F308^7.35^, Y316^7.43^, and F86^2.64^, which are primarily located in TM2, TM3, and TM7 (Fig. 3*C*). Among these residues, Q177^45.51^ and F86^2.64^ are less conserved within the α_1_AR family (Fig. S5). Consistent with our structural observations, previous mutagenesis studies demonstrated that F86^2.64^ critically mediates interactions between α_1A_AR and various antagonists, including HEAT and prazosin (27, 36, 37).

### Recognition mechanism of silodosin with α_1A_AR

Silodosin is a selective antagonist of the α_1A_AR subtype, exhibiting an affinity for α_1A_AR that is 162-fold greater than α_1B_AR and 50-fold greater than α_1D_AR (22). It belongs to the indolecarboxamide derivative class of drugs, containing an indole ring composed of fused pyrrole and benzene rings. The main chain of the ethyl-aminopropyl (R1) is substituted on both sides by indole carbamate groups (R2, R4) and a phenoxy group (R5), whereas the indole carbamate group is substituted by a hydroxylpropyl group (R3) and the phenoxy group is substituted by a trifluoroethoxy group (R6) (Fig. 3*A*) (33, 34).

The silodosin–α_1A_AR complex structure reveals that silodosin binds deeply within the orthosteric site of α_1A_AR, adopting an extended conformation and forming extensive interactions with surrounding residues (Fig. 3*A*, *D*–*F*). The phenoxy and trifluoroethoxy groups of silodosin penetrate deep into the binding pocket, forming extensive hydrophobic interactions with residues V107^3.33^, I178^45.52^, Y184^5.38^, V185^5.39^, S188^5.42^, M292^6.55^, F289^6.52^, F288^6.51^, and C110^3.36^ (Fig. 3*D*). In particular, the phenoxy group packs tightly against residues V107^3.33^ and F288^6.51^ and forms a π–π stacking interaction with F289^6.52^ (Fig. 3*D*). Among residues interacting with silodosin, M292^6.55^ and V185^5.39^ are particularly noteworthy, as they are uniquely present in α_1A_AR compared with other α_1_AR subtypes (Fig. S5). These distinctive residues likely contribute substantially to silodosin’s high selectivity toward α_1A_AR. At the opposite end, the indole carboxamide and hydroxypropyl groups of silodosin form nonpolar interactions with W313^7.40^, Y316^7.43^, W102^3.28^, I178^45.52^, G90^2.68^, F86^2.64^, and E87^2.65^, and a hydrogen bond with S83^2.61^ (Fig. 3*E*). Of these, F86^2.64^ and I178^45.52^ are 2 additional nonconserved residues that, together with M292^6.55^ and V185^5.39^, may underpin the selectivity of silodosin for α_1A_AR (Fig. S5). Finally, the ethyl-aminopropyl group in silodosin forms nonpolar interactions with C110^3.36^, W102^3.28^, W285^6.48^, and F288^6.51^, and its amine group participates in polar interactions with D106^3.32^ and Y316^7.43^ (Fig. 3*F*). In summary, silodosin is deeply integrated into the orthosteric site of α_1A_AR, and its selectivity is likely influenced by the presence of nonconserved residues within the binding pocket.

### Comparison of the ligand binding poses in the pocket

**Figure 4:**
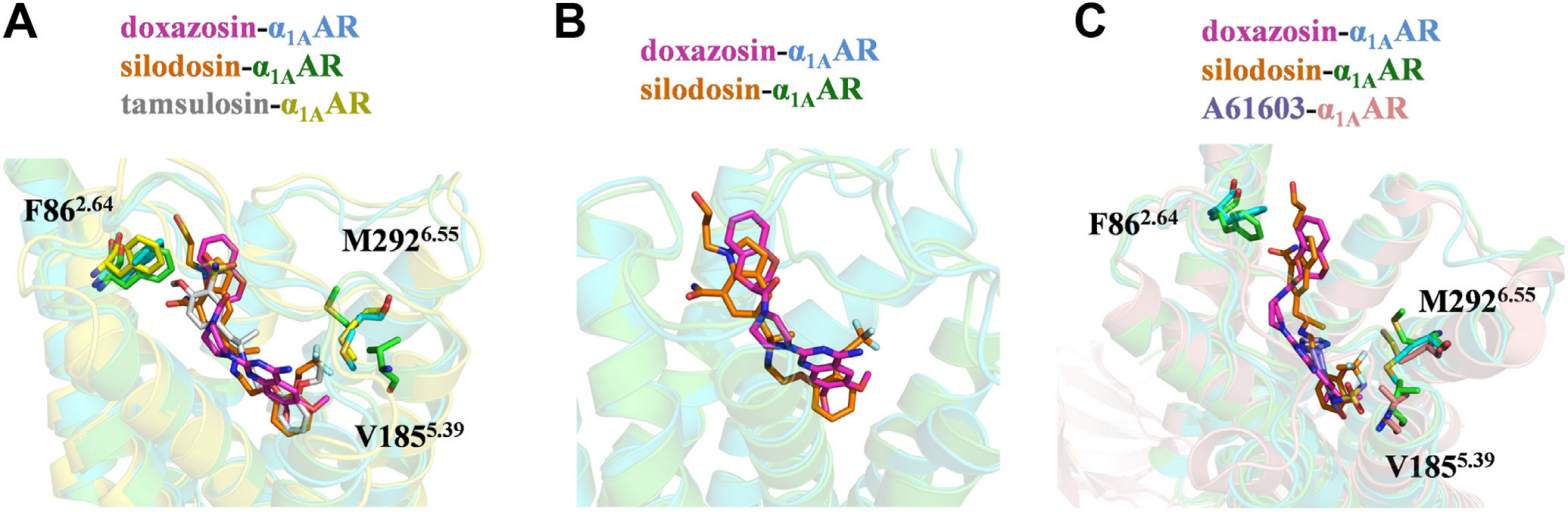
**Comparison of different ligand-binding pockets**. *A*, the binding differences in the binding pocket of α_1A_AR upon the interaction with doxazosin, silodosin, and tamsulosin (*light gray*) (PDB ID: 7YMJ). The α_1A_AR bound to tamsulosin is depicted in *yellow*. The phenyl groups of silodosin and tamsulosin penetrate more deeply into the orthosteric binding pocket. This figure highlights the interactions of 3 antagonists with the nonconserved residues F86^2.64^, V185^5.39^, and M292^6.55^. *B*, comparison of the binding poses of silodosin and doxazosin. Doxazosin, characterized by its rigid quinazoline ring, adopts a suboptimal fit within the pocket and is positioned closer to the receptor's outer region. *C*, the structural differences in the binding pocket of α_1A_AR following the interaction with doxazosin, silodosin, and A61603 (PDB ID: 8THK). This figure specifically illustrates interactions of the 3 ligands with nonconserved residues F86^2.64^, V185^5.39^, and M292^6.55^. PDB, Protein Data Bank.

To elucidate the recognition mechanism of α_1A_AR by its antagonists, we conducted a detailed comparison of ligand-binding poses and residue conformations within the orthosteric pocket when occupied by different antagonists. By aligning our structures with the previously reported tamsulosin–α_1A_AR complex (27), we observed that tamsulosin and silodosin adopt highly similar binding poses within the α_1A_AR orthosteric pocket (Fig. 4*A*). Both ligands position their phenyl moieties deeply within the receptor's hydrophobic pocket, whereas the ethyl-aminopropyl group and its associated derivatives extend toward the extracellular vestibule (Fig. 4*A*). In contrast, doxazosin, with its more rigid quinazoline ring, appears to have a suboptimal fit in the pocket, resulting in its positioning more toward the receptor's outer region (Fig. 4*B*). Consequently, doxazosin fails to establish the π–π interactions with F289^6.52^, which are observed for silodosin and tamsulosin (Fig. S6, *B–D*). In addition, van der Waals interactions involving residue E87^2.65^ were observed only for silodosin and tamsulosin but absent in doxazosin-bound complex (Fig. S6, *E–G*). Moreover, only silodosin forms an additional hydrogen bond with S83^2.61^ (Fig. S6, *E–G*). These differences likely account for the generally higher affinity of silodosin and tamsulosin for α_1A_AR compared with doxazosin (Fig. S6*A*).

Although silodosin and tamsulosin have comparable affinity for α_1A_AR (Fig. S6*A*), they exhibit markedly different selectivity profiles, silodosin being much more selective for α_1A_AR than tamsulosin (11, 22). Structural comparisons revealed that the trifluoroethoxy moiety of silodosin contributes to its α_1A_AR subtype selectivity. Particularly noteworthy are residues V185^5.39^ and M292^6.55^, which interact specifically with the trifluoroethoxy group (Fig. 4*A*). These residues are less conserved across α_1_AR subtypes and form conformations accommodating this substituent group (Figs. 4*A* and S5). Similar interactions involving V185^5.39^ and M292^6.55^ were also observed in the α_1A_AR selective ligand A61603-bound structure, further underscoring their important role in ligand discrimination (Fig. 4*C*). In addition, another nonconserved residue, F86^2.64^, located within the binding pocket, directly interacts with antagonists (Figs. 4*A* and S5). Interestingly, the orientation of F86^2.64^ varies slightly depending on the bound ligand, suggesting conformational flexibility in ligand recognition mechanisms (Fig. 4, *A* and *C*). Previous functional studies also indicated the importance of F86^2.64^ residue in recognition of diverse α_1A_AR antagonists including HEAT and prazosin (27, 36, 37). Therefore, F86^2.64^ likely plays an integral role in enabling effective recognition of silodosin and doxazosin by α_1A_AR.

### In-depth exploration of the selective mechanism of α_1A_AR with silodosin

**Figure 5:**
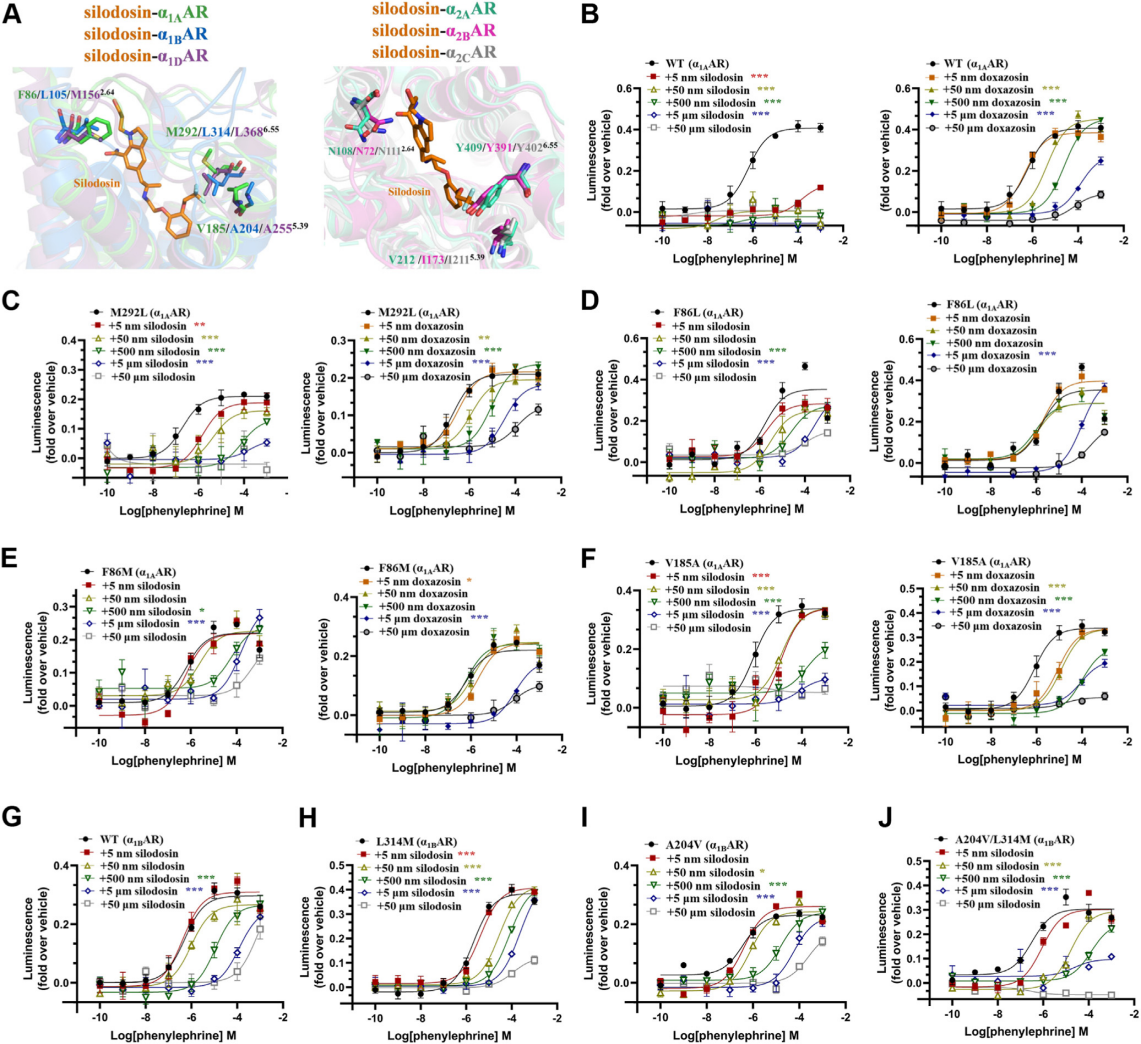
**In-depth exploration of silodosin subtype selectivity**. *A*, structural superposition of the silodosin–α_1A_AR complex with α_1B_AR (*dark blue*), α_1D_AR (*purple*), α_2A_AR (*cyan*), α_2B_AR (*pink*), and α_2C_AR (*light gray*). An overlay of α_1A_AR, α_1B_AR, and α_1D_AR is shown on the *left*, and an overlay of α_2A_AR, α_2B_AR, and α_2C_AR is shown on the *right*, with the nonconserved residues F86^2.64^, V185^5.39^, and M292^6.55^ highlighted. The PDB IDs for α_1B_AR, α_2A_AR, and α_2C_AR are 7B6W, 6KUX, and 6KUW, respectively. Structures of α_1D_AR and α_2B_AR were predicted using AlphaFold2. *B*–*F*, the inhibitory effects of varying concentrations of silodosin (*left*) and doxazosin (*right*) on wildtype α_1A_AR as well as α_1A_AR mutants M292L, F86L, F86M, and V185A, which were assessed through NanoBiT recruitment experiments. *G*–*J*, the inhibitory effects of different concentrations of silodosin on wildtype α_1B_AR and α_1B_AR mutants L314M, A204V, and A204V/L314M, which were evaluated through NanoBiT recruitment experiments. *B*–*J*, luminescence data (fold over vehicle), averaged from NanoBiT recruitment assays (n = 3), were used as input for plotting. Agonist activation curves for phenylephrine were then fitted using a three-parameter logistic equation. Activation curves are shown for various concentrations of antagonist, differentiated by color and shape. Activation curves in the absence of antagonist are indicated in *black*. Data are presented as mean ± SD. Statistically significant differences between activation curves in the presence and absence of a specific concentration of antagonist were determined by comparing the -LogEC_50_ values obtained from 3 separately fitted curves using one-way ANOVA, followed by Dunnett's multiple comparison test (∗*p* < 0.05, ∗∗*p* < 0.01, and ∗∗∗*p* < 0.001). Due to inadequate activation of several experimental groups at an antagonist concentration of 50 μM, these data were omitted from subsequent statistical analysis to maintain uniformity.

Guided by structural insights, we next sought to clarify the molecular mechanism underlying the subtype selectivity of silodosin for α_1A_AR. We superimposed the silodosin-bound α_1A_AR structure onto those of α_1B_AR, α_1D_AR, α_2A_AR, α_2B_AR, and α_2C_AR, utilizing either experimentally determined structures or AlphaFold2-predicted models. Structural alignment revealed that while silodosin fits well with residue M292^6.55^ in α_1A_AR, it would result in steric clashes with residue L^6.55^ in α_1B_AR and α_1D_AR (Fig. 5*A*). Similarly, residue Y^6.55^ in α_2A_AR, α_2B_AR, and α_2C_AR would also obstruct silodosin binding (Fig. 5*A*). To validate these structural observations, we mutated α_1A_AR residue M292^6.55^ to either L^6.55^ or Y^6.55^, matching those found in α_1B_AR/α_1D_AR or α_2A_AR/α_2B_AR/α_2C_AR, respectively, and tested the mutants using the NanoBiT recruitment assay (Figs. 5*C*, Fig. S8*A* and S7). As anticipated, both substitutions (L^6.55^ and Y^6.55^) significantly impaired the ability of silodosin to inhibit phenylephrine-induced activation of α_1A_AR (Figs. 5, *B* and *C*, Fig. S8*A*, S9, *A* and *C* and Fig. S10, *A* and *H*). Conversely, the inhibitory activity of doxazosin was not diminished by these substitutions (Figs. 5, *B* and *C*, Fig. S8*A*, S9, *A* and *D* and Fig. S10, *A* and *I*), highlighting the crucial role of M292^6.55^ in determining ligand selectivity. Furthermore, introducing a methionine substitution at position L314^6.55^ in α_1B_AR substantially enhanced the antagonistic potency of silodosin toward the modified α_1B_AR receptor (Figs. 5, *G* and *H* and Fig. S9, *B* and *E*). In contrast, the same L314^6.55^ substitution did not improve the inhibitory potency of doxazosin (Figs. S8, *D*–*E* and Fig. S10, *D*, *E* and *J*).

Next, we assessed the contribution of residue F86^2.64^ to α_1A_AR ligand selectivity (Fig. 5*A*). In contrast to mutations at position M292^6.55^, substitutions of F86^2.64^ with equivalent residues from α_1B_AR, α_1D_AR, α_2A_AR, α_2B_AR, or α_2C_AR substantially decreased the inhibitory efficacy of both silodosin and doxazosin (Fig. 5*B*, *D*–*E*, Figs. S8*B*, S9*A*, *C*–*D* and Fig. S10*B*, *H*–*I*). This indicates that F86^2.64^ functions as a pivotal residue necessary for ligand engagement, but it plays a less specific role in dictating subtype-selective ligand binding.

Finally, we examined residue V185^5.39^ because of its unique presence in α_1A_AR (Figs. 5*A* and S5). Similarly, swapped constructs were generated and assessed for their responsiveness to silodosin. Consistent with observations for M292^6.55^, substituting V185^5.39^ in α_1A_AR with the equivalent residues from α_1B_AR, α_1D_AR, α_2A_AR, α_2B_AR, or α_2C_AR significantly reduced silodosin's inhibitory activity but did not reduce the inhibitory potency of doxazosin (Fig. 5, *B* and *F*, Figs. S8*C*, S9*A*, *C*–*D* and Fig. S10*C*, *H*–*I*). Importantly, simultaneous conversion of L314^6.55^ and A204^5.39^ in α_1B_AR to those found in α_1A_AR markedly enhanced silodosin’s antagonistic activity but did not enhance the inhibitory potency of doxazosin (Figs. 5, *G* and *J*, Fig. S8, *D* and *G*, Fig. S9, *B* and *E* and Fig. S10, *D*, *G* and *J*), further confirming V185^5.39^ as another determinant of α_1A_AR subtype selectivity. In sum, together with the structural and biochemical analyses, we demonstrate that V185^5.39^ and M292^6.55^ of α_1A_AR determined the receptor selectivity with silodosin.

## Discussion

The development of α_1_AR antagonists for the treatment of BPH has progressed from nonselective α-adrenoceptor antagonists to selective α_1_AR antagonists and subsequently to subtype-selective α_1A_AR antagonists. However, these drugs still exhibit certain side effects, highlighting the necessity for the development of new pharmaceuticals with reduced side effects and enhanced efficacy. This study elucidates the complex structures of the α_1_AR selective antagonist doxazosin and the α_1A_AR subtype–selective antagonist silodosin with α_1A_AR, resolved at 2.99 Å and 3.19 Å, respectively. Structural analysis suggests that the trifluoroethoxy group of silodosin may contribute to its subtype selectivity through interactions with nonconserved residues V185^5.39^ and M292^6.55^. Furthermore, superimposition of the silodosin-bound α_1A_AR structure onto those of other receptor subtypes reveals that L/Y^6.55^ creates varying degrees of steric hindrance, potentially influencing its binding affinity to different receptors. Building on structural analysis, we conducted mutagenesis functional assays, which demonstrated that M292^6.55^, V185^5.39^, and F86^2.64^ play crucial roles during the binding of silodosin to α_1A_AR. Specifically, M292^6.55^ and V185^5.39^ are essential residues conferring subtype selectivity to silodosin. Furthermore, we modified α_1B_AR, discovering that silodosin exhibited significantly enhanced inhibitory capacity against its A204V/L314M double mutant, further corroborating the critical roles of M292^6.55^ and V185^5.39^ in the subtype selectivity of silodosin.

This research provides an in-depth discussion of the molecular mechanisms by which antagonists recognize and regulate α_1A_AR, and validates the molecular principles governing the selective binding of silodosin to α_1A_AR, thereby offering more effective insights for the development of α_1A_AR subtype–selective antagonists. Furthermore, we noted in previous studies that M292^6.55^ and V185^5.39^ are pivotal residues that contribute to subtype selectivity in the agonist A61603 (28). This suggests that existing high subtype-selective drugs for α_1A_AR enhance selectivity through similar mechanisms. Whether alternative strategies can further improve selectivity remains a topic for future exploration. In summary, our research provides a robust structural foundation for the development of novel subtype-selective drugs targeting α_1A_AR and lays the groundwork for future investigations into other GPCRs.

## Experimental procedures

### Expression and purification of Fab and glue molecules

A pelB signal peptide was added to the N terminus of the Fab and glue molecular sequences, whereas a His tag was incorporated at the C terminus (Data S4) (29). This sequence was subsequently ligated into the pET-22b (+) vector (29). The recombinant expression vector was transformed into *Escherichia coli* BL21 (DE3) cells, and colonies were selected for expansion in liquid LB medium containing ampicillin. Following this, IPTG was added to induce expression at a final concentration of 0.5 mM. Cells were harvested, subjected to low-temperature ultrasonic disruption, and purified using nickel–nitrilotriacetic acid chromatography. Wash buffer (20 mM imidazole, 150 mM NaCl, 20 mM Hepes [pH 7.5]) was employed to remove impurities. The target protein was eluted using elution buffer (250 mM imidazole, 150 mM NaCl, 20 mM Hepes [pH 7.5]). The Fab protein was concentrated using a 30 kDa molecular weight cutoff concentrator (Millipore), whereas the glue protein was concentrated using a 10 kDa concentration tube. The proteins were then aliquoted and stored in a refrigerator at 4 °C for future use.

### Construct design of α_1A_AR–mBRIL

The gene sequence of the α_1A_AR–mBRIL fusion receptor was constructed by modifying the gene sequence of the human α_1A_AR. The α_1A_AR–mBRIL fusion receptor utilizes a hemagglutinin signal peptide (hemagglutinin sequence: MKTIIALSYIFCLVFA) in place of the original signal peptide and incorporates FLAG (FLAG sequence: DYKDDDDK) and His tags for subsequent protein purification (Data S4) (29). The mBRIL sequence is inserted between TM5 and TM6 of the α_1A_AR, and a K3-ALFA tag is introduced following helix 8 of the receptor, culminating in the incorporation of this sequence into the PSCST mammalian expression vector (Data S4) (29). The PSCST vector has resistance to ampicillin and has been modified from the pCDNA3.1 vector (29).

### Complex formation and purification

Expi293F cells (Thermo Fisher; A14527) were used to express the α_1A_AR–mBRIL fusion receptor, and transfection was performed using polyethyleneimine (Polysciences; catalog no.: 23966) reagents. Following a 60-h transfection period, the cells were harvested and stored at −20 °C for later use. To the thawed cells, low-salt lysis buffer was added, which included 3 μM doxazosin (antagonist TOPSCIENCE; catalog no.: T22316) or 1 μM silodosin (antagonist TOPSCIENCE; catalog no.: T1504), 0.5 mM EDTA, 1 mM PMSF, and 10 mM Hepes at pH 7.5. The mixture was homogenized and stirred at a constant rate for 30 min at 4 °C. After centrifugation, the resulting pellet was resuspended in a membrane buffer containing 3 μM doxazosin or 1 μM silodosin, 1% (w/v) *n*-dodecyl-β-d-maltoside (Anatrace; catalog no.: D310), 500 mM NaCl, 0.1% (w/v) cholesteryl hemisuccinate (Sigma; catalog no.: C6512), and 20 mM Hepes at pH 7.5, followed by grinding of the pellet. Subsequently, the mixture was incubated with nickel–nitrilotriacetic acid chromatography at 4 °C for 2 h. The resin was collected in a chromatography column, and wash buffer containing 10 mM imidazole was added to remove impurities. The protein was then eluted using elution buffer containing 250 mM imidazole. An excess of glue protein and Fab protein, anti-FLAG M1 affinity resin (M1 resin; Sigma–Aldrich; catalog no.: A4596), and a final concentration of 2 mM CaCl_2_ solution were added to the eluted protein solution, followed by incubation at 4 °C for 1 h. The M1 resin was collected, and the detergent in the solution was gradually replaced with LMNG. Thereafter, elution buffer containing 3 μM doxazosin or 1 μM silodosin, 0.00025% (w/v) glycol–diosgenin (Anatrace; catalog no.: GDN101), 0.00075% (w/v) LMNG, 0.0001% (w/v) cholesteryl hemisuccinate, 100 μg/ml FLAG peptide, 150 mM NaCl, 5 mM EDTA, and 20 mM Hepes at pH 7.5 was used to elute the protein. Size exclusion chromatography was conducted using a pre-equilibrated Superdex 200 Increase 10/300 column (GE Healthcare), which had been treated with the equilibration solution in advance. The protein from monodisperse peak fractions was concentrated to 6 to 8 mg/ml and stored on ice for the preparation of cryo-EM samples.

### Cryo-EM sample preparation and data acquisition

The sample preparation parameters were set as follows: humidity was configured at 100%, temperature at 8 °C, blot time was established at 4 s, blot force was designated as 1, waiting time was fixed at 4 s, and blot total was determined to be 1. The sample preparation apparatus was assembled, and hydrophilization treatment of the glow-charged amorphous alloy film grid (CryoMatrix nickel titanium alloy film, R1.2/1.3; Zhenjiang Lehua Electronic Technology Co, Ltd) was conducted to facilitate its adsorption of the sample (38). The carrier grid was secured onto the Vitrobot Mark IV (Thermo Fisher Scientific). A 4 to 5 μl aliquot of the protein solution was dispensed onto the carrier grid, followed by a waiting period for the machine to execute the fully automated subsequent processes. The samples were carefully transferred to a grid box and stored long term in a liquid nitrogen tank. Images were collected using a 300-kV Titan Krios Gi3 microscope (Thermo Fisher Scientific FEI, the Center for Integrative Imaging, Hefei National Laboratory for Physical Sciences at the Microscale, University of Science and Technology of China) operating at 300 kV, utilizing a GIF energy filter (Gatan), with a magnification of 81,000 times. Each image captured 32 frames, with a total exposure time of 2 s, an electron dose rate of 35.09 e/pixel/s, and the Counted Super Resolution mode was employed, with an underfocus value ranging from −1.7 to −2.3 μm.

### Cryo-EM data processing

For the doxazosin–α_1A_AR and silodosin–α_1A_AR complexes, we collected 3417 and 3402 images, respectively. After correction of the beam-induced motion by MotionCor2 (University of California) (39), the micrographs were imported into cryoSPARC (Structura Biotechnology Inc) (40), and contrast transfer function parameters were estimated by Patch CTF. 3,997,625 and 3,759,439 particles were extracted with a pixel size of 1.652 Å after autopicking by Template Picker. Two rounds of 2D classification, two rounds of initial model generation, and two rounds of heterogeneous refinement were performed in cryoSPARC. The obtained 787,717 and 632,032 particles were re-extracted in RELION (MRC Laboratory of Molecular Biology) (41, 42) with a pixel size of 0.826 Å. After 3D autorefinement in RELION, the particles were further sieved using CryoSieve (Tsinghua University) (43), then, 132,158 and 132,548 particles were retained. The selected particles were finally applied to Polish in RELION and local refinement in cryoSPARC, generating a density map of 2.99 Å and 3.19 Å resolution.

### Model building and refinement

The structure of the α_1A_AR fused with mBRIL was predicted using AlphaFold2, serving as the initial model for building the model. The structure of E3 and K3 within the complex is derived from the E3/K3 coiled-coil (Protein Data Bank [PDB] ID: 1U0I). ALFA and ALFA-Nb are sourced from the crystal structure of the ALFA-tag binding nanobody (PDB ID: 6I2G). The Fab–NbFab within the complex originates from the crystal structure of pinatuzumab Fab in complex with the anti-Kappa VHH domain (PDB ID: 6AND) (29). The model was docked into the electron density map using Chimera (UCSF Resource for Biocomputing, Visualization, and Informatics) (44), followed by manual adjustments in COOT (MRC Laboratory of Molecular Biology) (45). Subsequently, the model was further refined using Phenix (Lawrence Berkeley National Laboratory) (46, 47), and the resulting structure required additional adjustments in COOT, continuing in a cyclical manner until a high correspondence between the structural model and the electron density was attained. The statistical parameters of the final model were validated using MolProbity (Duke University) (48), and the summary is presented in Table S1. Structural analysis and result visualization were performed using software such as PyMOL (The PyMOL Molecular Graphics System, Version 3.1) and UCSF Chimera (49).

### Nanobit G protein recruitment assay

This study aims to validate functional experiments using Nanobit G protein recruitment assays (50). The receptor is fused with Large BiT (LgBiT), whereas Gβ is fused with Small BiT (SmBiT). If the target protein can interact, the 2 tags complementarily form luciferase, which emits fluorescence upon the addition of the substrate furimazine (Fig. S7*A*) (50).

Human embryonic kidney 293T cells (Pricella; CL-0005), seeded in 6-well plates (Corning), were transfected using Lipo8000 (Beyotime; C0533) with 500 ng of the GPCR-LgBiT expression vector, 1 μg of Gαq, 500 ng of SmBiT-Gβ, and 500 ng of Gγ recombinant expression vectors. The amino acid sequences for α_1A_AR-LgBiT, α_1B_AR-LgBiT, Gαq, SmBit-Gβ, and Gγ are detailed in Data S4 (50). About 24 h post-transfection, cells were harvested and resuspended in Dulbecco's modified Eagle's medium (DMEM) (Hyclone). In a white, 96-well cell culture plate (Beyotime), 80 μl of 293T cell suspension, 10 μl of antagonist at specific concentrations (12×, diluted in DMEM), and 20 μl of 5 μM substrate (6×, diluted in DMEM) were sequentially added. Following a 15 min incubation at room temperature, background luminescent signals were measured using a luminescent microplate reader (SpectraMax iD5; Molecular Devices). Subsequently, 10 μl of agonist at varying concentrations (12×, diluted in DMEM) was added, alongside 10 μl of DMEM without agonist as a vehicle control. Following an additional 15-min incubation at room temperature, luminescence signals were measured again. Raw luminescence signals were normalized by first dividing each measurement by its corresponding background luminescent signal and then subtracting the luminescence value obtained from control wells (without agonist). This yielded the relative luminescence responses (fold over vehicle). The resulting luminescence (fold over vehicle) data were then fitted using the log(agonist) *versus* response (3 parameters) equation in GraphPad Prism 8 (GraphPad Software, Inc) to generate activation curves for each antagonist concentration. By comparing the trends in activation effects for wildtype receptors and mutants in the presence of different antagonist concentrations, the impact of various mutations on the inhibitory effects of silodosin and doxazosin was determined (Fig. S7*B*) (50).

### Statistical analyses

Statistical analyses were performed in R (v 4.4.2). Dunnett's test (51, 52) was conducted using the DescTools package (v 0.99.59, https://andrisignorell.github.io/DescTools/). Error bars represent standard deviations calculated from 3 replicates. Detailed statistical differences and quantitative descriptions are presented in the figure legends. Statistical data are available in Data S1–S3.

To provide a quantitative description of changes in activation efficacy (quantified using EC_50_) with increasing antagonist concentration, we incorporated statistical analysis of activation curves (Figs. S9, *A*–*B* and Fig. S10, *A*–*G*) and reported detailed results including mean difference, confidence intervals, and *p* value (Data S1–S3). We also compared the differences in change between various mutants and the wildtype receptor under the same antagonist concentration (Figs. S9, *C*–*E*, Fig. S10, *H*–*J*). For example, in Figure S9*D* and its corresponding bar graphs, the wildtype α_1A_AR exhibited a significant decrease starting at 50 nM (mean difference: −0.84; fold change: 0.87, *p* < 0.001), whereas F86M showed a highly significant decrease only at 5 μM (mean difference: −2.12; fold change: 0.66, *p* < 0.001). In contrast, V185A displayed a significant decrease as early as 5 nM (mean difference: −0.97; fold change: 0.84, *p* < 0.001); thus, a clear difference exists between the wildtype α_1A_AR and the 2 mutants. The changes in the ability of doxazosin to inhibit receptor activation are presented as mean difference and fold change (Data S1–S3).

## Data availability

The structural data obtained in this study have been deposited in the PDB. The 3D cryo-EM maps have been deposited in the Electron Microscopy Data Bank (EMDB). The PDB ID for doxazosin–α_1A_AR is 9M4Q and the EMDB ID is EMD-63628. The PDB ID for silodosin–α_1A_AR is 9M4T and the EMDB ID is EMD-63629. PDB data used in this study include 8THK, 7B6W, 1U0I, 6I2G, 6AND, 7YMJ, 6KUX, and 6KUW.

## Supporting information

This article contains supporting information, in which the Guide to Pharmacology (https://www.guidetopharmacology.org/) is cited as a web resource.

## Conflict of interest

The authors declare that they have no conflicts of interest with the contents of this article.

## References

[1] Archer M., Dogra N., Dovey Z., Ganta T., Jang H.S., Khusid J.A. (2021). Role of α- and β-adrenergic signaling in phenotypic targeting: significance in benign and malignant urologic disease. Cell. Commun. Signal..

[2] Ahlquist R.P. (1948). A study of the adrenotropic receptors. Am. J. Physiol..

[3] Bylund D.B., Eikenberg D.C., Hieble J.P., Langer S.Z., Lefkowitz R.J., Minneman K.P. (1994). International union of pharmacology nomenclature of adrenoceptors. Pharmacol. Rev..

[4] Graham R.M., Perez D.M., Hwa J., Piascik M.T. (1996). Alpha 1-adrenergic receptor subtypes. Molecular structure, function, and signaling. Circ. Res..

[5] Akinaga J., Garcia-Sainz J.A., A S.P. (2019). Updates in the function and regulation of alpha(1) -adrenoceptors. Br. J. Pharmacol..

[6] Perez D.M. (2023). alpha(1)-Adrenergic receptors: insights into potential therapeutic opportunities for COVID-19, heart failure, and Alzheimer's disease. Int. J. Mol. Sci..

[7] (2012). Alpha 1 adrenergic receptor antagonists in LiverTox: Clinical and research Information on drug-induced liver injury.

[8] Hilal-Dandan R., Brunton L.L. (2016). Goodman and Gilman's Manual of Pharmacology and Therapeutics, 2e.

[9] (2003). Diuretic versus alpha-blocker as first-step antihypertensive therapy: final results from the antihypertensive and lipid-lowering treatment to prevent heart attack trial (ALLHAT). Hypertension.

[10] James P.A., Oparil S., Carter B.L., Cushman W.C., Dennison-Himmelfarb C., Handler J. (2014). 2014 evidence-based guideline for the management of high blood pressure in adults: report from the panel members appointed to the Eighth Joint National Committee (JNC 8). JAMA.

[11] Lepor H. (2007). Alpha blockers for the treatment of benign prostatic hyperplasia. Rev. Urol..

[12] Caine M., Pfau A., Perlberg S. (1976). The use of alpha-adrenergic blockers in benign prostatic obstruction. Br. J. Urol..

[13] Caine M., Perlberg S., Meretyk S. (1978). A placebo-controlled double-blind study of the effect of phenoxybenzamine in benign prostatic obstruction. Br. J. Urol..

[14] Kirby R.S., Coppinger S.W., Corcoran M.O., Chapple C.R., Flannigan M., Milroy E.J. (1987). Prazosin in the treatment of prostatic obstruction. A placebo-controlled study. Br. J. Urol..

[15] Kirby R.S. (1998). Terazosin in benign prostatic hyperplasia: effects on blood pressure in normotensive and hypertensive men. Br. J. Urol..

[16] Kirby R.S. (1995). Doxazosin in benign prostatic hyperplasia: effects on blood pressure and urinary flow in normotensive and hypertensive men. Urology.

[17] van Kerrebroeck P., Jardin A., Laval K.U., van Cangh P. (2000). Efficacy and safety of a new prolonged release formulation of alfuzosin 10 mg once daily versus alfuzosin 2.5 mg thrice daily and placebo in patients with symptomatic benign prostatic hyperplasia. ALFORTI Study Group. Eur. Urol..

[18] Roehrborn C.G. (2001). Efficacy and safety of once-daily alfuzosin in the treatment of lower urinary tract symptoms and clinical benign prostatic hyperplasia: a randomized, placebo-controlled trial. Urology.

[19] Kenny B.A., Miller A.M., Williamson I.J., O'Connell J., Chalmers D.H., Naylor A.M. (1996). Evaluation of the pharmacological selectivity profile of alpha 1 adrenoceptor antagonists at prostatic alpha 1 adrenoceptors: binding, functional and in vivo studies. Br. J. Pharmacol..

[20] Forray C., Bard J.A., Wetzel J.M., Chiu G., Shapiro E., Tang R. (1994). The alpha 1-adrenergic receptor that mediates smooth muscle contraction in human prostate has the pharmacological properties of the cloned human alpha 1c subtype. Mol. Pharmacol..

[21] Yoshida M., Kudoh J., Homma Y., Kawabe K. (2011). Safety and efficacy of silodosin for the treatment of benign prostatic hyperplasia. Clin. Interv. Aging..

[22] Tatemichi S., Kobayashi K., Maezawa A., Kobayashi M., Yamazaki Y., Shibata N. (2006). [Alpha1-adrenoceptor subtype selectivity and organ specificity of silodosin (KMD-3213)]. Yakugaku. Zasshi..

[23] (2009). Silodosin (Rapaflo) for benign prostatic hyperplasia. Med. Lett. Drugs. Ther..

[24] Yang D., Zhou Q., Labroska V., Qin S., Darbalaei S., Wu Y. (2021). G protein-coupled receptors: structure- and function-based drug discovery. Signal. Transduct. Target. Ther..

[25] Congreve M., de Graaf C., Swain N.A., Tate C.G. (2020). Impact of GPCR structures on drug discovery. Cell.

[26] Ballante F., Rudling A., Zeifman A., Luttens A., Vo D.D., Irwin J.J. (2020). Docking finds GPCR ligands in dark chemical matter. J. Med. Chem..

[27] Toyoda Y., Zhu A., Kong F., Shan S., Zhao J., Wang N. (2023). Structural basis of α1A-adrenergic receptor activation and recognition by an extracellular nanobody. Nat. Commun..

[28] Su M., Wang J., Xiang G., Do H.N., Levitz J., Miao Y. (2023). Structural basis of agonist specificity of α1A-adrenergic receptor. Nat. Commun..

[29] Guo Q., He B., Zhong Y., Jiao H., Ren Y., Wang Q. (2024). A method for structure determination of GPCRs in various states. Nat. Chem. Biol..

[30] Zhou Q., Yang D., Wu M., Guo Y., Guo W., Zhong L. (2019). Common activation mechanism of class A GPCRs. Elife.

[31] Liu X., Ahn S., Kahsai A.W., Meng K.C., Latorraca N.R., Pani B. (2017). Mechanism of intracellular allosteric β(2)AR antagonist revealed by X-ray crystal structure. Nature.

[32] Ballesteros J.A., Weinstein H., Sealfon S.C. (1995). Methods in Neurosciences.

[33] Kim S., Chen J., Cheng T., Gindulyte A., He J., He S. (2025). PubChem 2025 update. Nucleic. Acids. Res..

[34] Knox C., Wilson M., Klinger C.M., Franklin M., Oler E., Wilson A. (2024). DrugBank 6.0: the DrugBank Knowledgebase for 2024. Nucleic Acids Res..

[35] Strader C.D., Sigal I.S., Register R.B., Candelore M.R., Rands E., Dixon R.A. (1987). Identification of residues required for ligand binding to the beta-adrenergic receptor. Proc. Natl. Acad. Sci. U. S. A..

[36] Hamaguchi N., True T.A., Goetz A.S., Stouffer M.J., Lybrand T.P., Jeffs P.W. (1998). Alpha 1-adrenergic receptor subtype determinants for 4-piperidyl oxazole antagonists. Biochemistry.

[37] Hamaguchi N., True T.A., Saussy D.L., Jeffs P.W. (1996). Phenylalanine in the second membrane-spanning domain of alpha 1A-adrenergic receptor determines subtype selectivity of dihydropyridine antagonists. Biochemistry.

[38] Huang X., Zhang L., Wen Z., Chen H., Li S., Ji G. (2020). Amorphous nickel titanium alloy film: a new choice for cryo electron microscopy sample preparation. Prog. Biophys. Mol. Biol..

[39] Zheng S.Q., Palovcak E., Armache J.P., Verba K.A., Cheng Y., Agard D.A. (2017). MotionCor2: anisotropic correction of beam-induced motion for improved cryo-electron microscopy. Nat. Methods.

[40] Punjani A., Rubinstein J.L., Fleet D.J., Brubaker M.A. (2017). cryoSPARC: algorithms for rapid unsupervised cryo-EM structure determination. Nat. Methods..

[41] Fernandez-Leiro R., Scheres S.H.W. (2017). A pipeline approach to single-particle processing in RELION. Acta. Crystallogr. D. Struct. Biol..

[42] Scheres S.H. (2012). RELION: implementation of a Bayesian approach to cryo-EM structure determination. J. Struct. Biol..

[43] Zhu J., Zhang Q., Zhang H., Shi Z., Hu M., Bao C. (2023). A minority of final stacks yields superior amplitude in single-particle cryo-EM. Nat. Commun..

[44] Pettersen E.F., Goddard T.D., Huang C.C., Couch G.S., Greenblatt D.M., Meng E.C. (2004). UCSF Chimera--a visualization system for exploratory research and analysis. J. Comput. Chem..

[45] Emsley P., Lohkamp B., Scott W.G., Cowtan K. (2010). Features and development of coot. Acta. Crystallogr. D. Biol. Crystallogr..

[46] Adams P.D., Afonine P.V., Bunkóczi G., Chen V.B., Davis I.W., Echols N. (2010). PHENIX: a comprehensive Python-based system for macromolecular structure solution. Acta. Crystallogr. D. Biol. Crystallogr..

[47] Liebschner D., Afonine P.V., Baker M.L., Bunkóczi G., Chen V.B., Croll T.I. (2019). Macromolecular structure determination using X-rays, neutrons and electrons: recent developments in Phenix. Acta Crystallogr. D Struct. Biol..

[48] Chen V.B., Arendall W.B., Headd J.J., Keedy D.A., Immormino R.M., Kapral G.J. (2010). MolProbity: all-atom structure validation for macromolecular crystallography. Acta. Crystallogr. D. Biol. Crystallogr..

[49] Goddard T.D., Huang C.C., Meng E.C., Pettersen E.F., Couch G.S., Morris J.H. (2018). UCSF ChimeraX: meeting modern challenges in visualization and analysis. Protein. Sci..

[50] Inoue A., Raimondi F., Kadji F.M.N., Singh G., Kishi T., Uwamizu A. (2019). Illuminating G-protein-coupling selectivity of GPCRs. Cell.

[51] Dunnett C.W. (1955). A multiple comparison procedure for comparing several treatments with a control. J. Am. Stat. Assoc..

[52] Michel M.C., Murphy T.J., Motulsky H.J. (2020). New author guidelines for displaying data and reporting data analysis and statistical methods in experimental biology. J. Pharmacol. Exp. Ther..

