# Supplementary material for: Molecular mechanism of antagonist recognition and regulation of the α1A-adrenoceptor
Source: J Biol Chem. 2025 Jun 6;301(7):110348. doi: 10.1016/j.jbc.2025.110348 (PMC12269490; doi:10.1016/j.jbc.2025.110348)
Supplement: Supporting Information [file mmc1.pdf]

**Supporting Information for**

# Molecular mechanism of antagonists recognition and regulation of the $\alpha_{1A}$-adrenoceptor.

($\alpha_{1A}$-Adrenoceptor Antagonist Recognition)

Sisi Liu[1], Haizhan Jiao[2], Yuyong Tao[1*], Dandan Wang[1*], Qiong Guo[1*]

1. Department of Laboratory Medicine, The First Affiliated Hospital of USTC, MOE Key Laboratory for Membraneless Organelles and Cellular Dynamics, Hefei National Center for Cross-disciplinary Sciences, Biomedical Sciences and Health Laboratory of Anhui Province, Center for Advanced Interdisciplinary Science and Biomedicine of IHM, Division of Life Sciences and Medicine, University of Science and Technology of China, Hefei, China.

2. Kobilka Institute of Innovative Drug Discovery, School of Medicine, The Chinese University of Hong Kong, Shenzhen, China.

**The PDF file includes:**

Figs. S1 to S10

Table S1

**Other Supporting Information for this manuscript include the following:**

Movies S1 to S2

Data S1 to S4

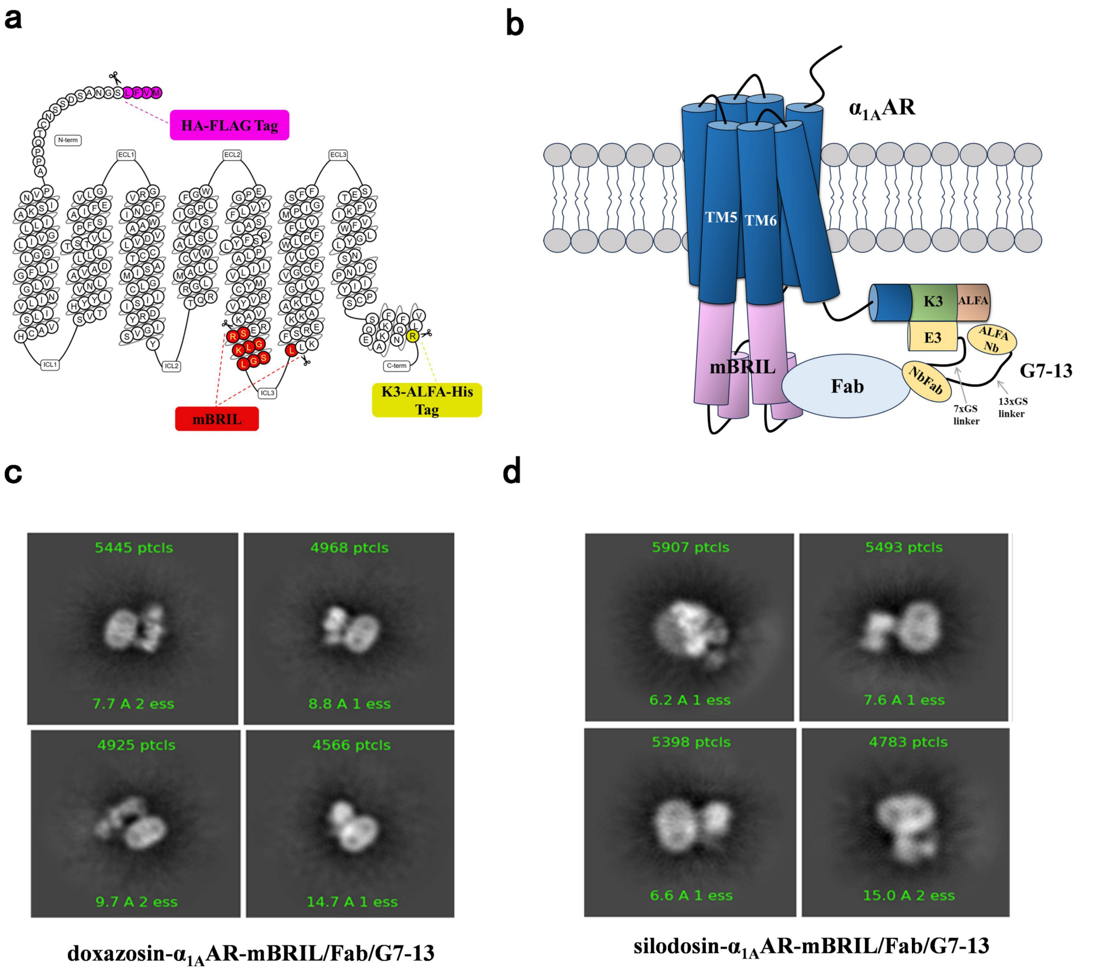


**Fig. S1: Plasmid design, assembly of the complex, and representative 2D classifications of the two complexes.**

**a.** Plasmid design for the $\alpha_{1A}$AR-mBRIL fusion receptor. Insertion sites for the HA-FLAG tag (pink), mBRIL (red), and K3-ALFA-His tag (yellow) are indicated. **b.** Schematic representation of the $\alpha_{1A}$AR-mBRIL/Fab/G7-13 complex assembly. $\alpha_{1A}$AR, mBRIL, ALFA, and K3 are represented as dark blue, pink, orange, and green cylinders, respectively. Fab is shown as a light blue oval. ALFANb, NbFab, and E3 in G7-13 are depicted in yellow. The 7×GS linker and 13×GS linker are indicated by text labels. **c-d.** Representative 2D classifications of the two complexes.

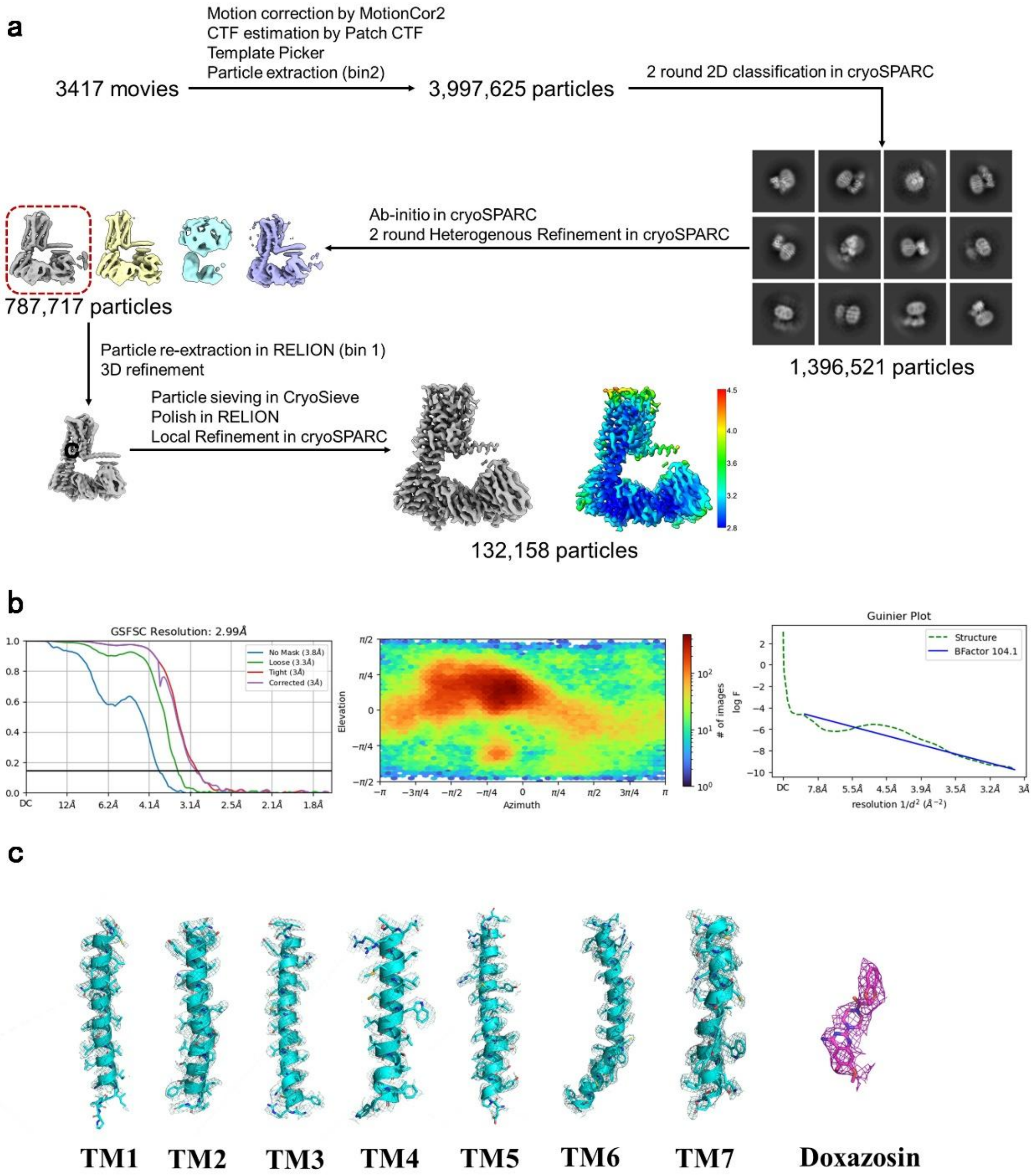


**Fig. S2: Cryo-EM processing and 3D reconstruction workflow for the doxazosin-$\alpha_{1A}$AR complex.**

**a.** Cryo-EM data processing workflow and local resolution map of doxazosin-$\alpha_{1A}$AR complex. **b.** The Gold-standard FSC curves of the doxazosin-$\alpha_{1A}$AR complex 3D reconstructions. **c.** The structural model of the doxazosin-$\alpha_{1A}$AR complex compared with the cryo-EM map, with cryo-EM map depicted in light blue and pink.

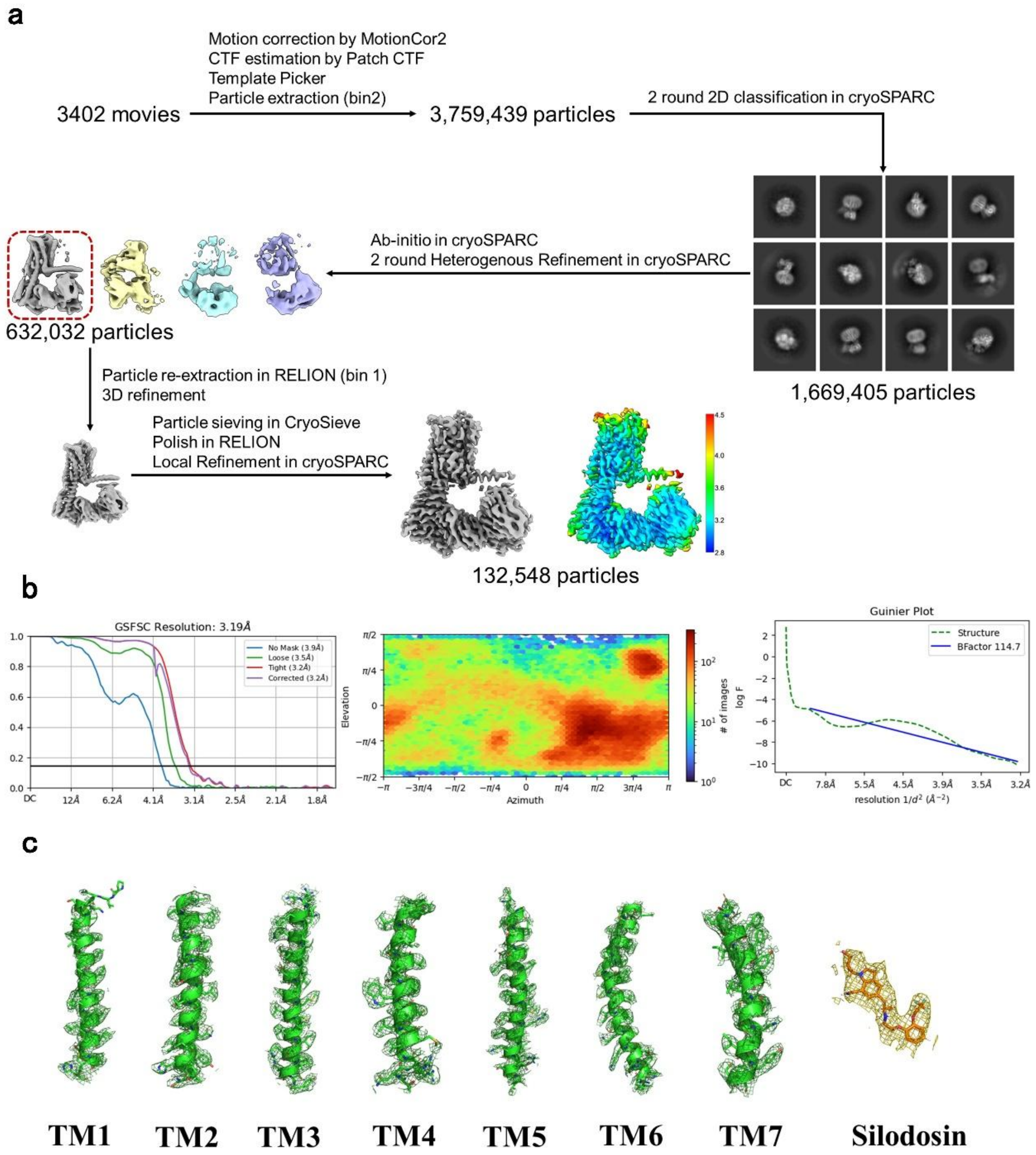


**Fig. S3: Cryo-EM processing and 3D reconstruction workflow for the silodosin-$\alpha_{1A}$AR complex.**

**a.** Cryo-EM data processing workflow and local resolution map of silodosin-$\alpha_{1A}$AR complex. **b.** The Gold-standard FSC curves of the silodosin-$\alpha_{1A}$AR complex 3D reconstructions. **c.** The structural model of the silodosin-$\alpha_{1A}$AR complex compared with the cryo-EM map, with cryo-EM map depicted in light green and orange.

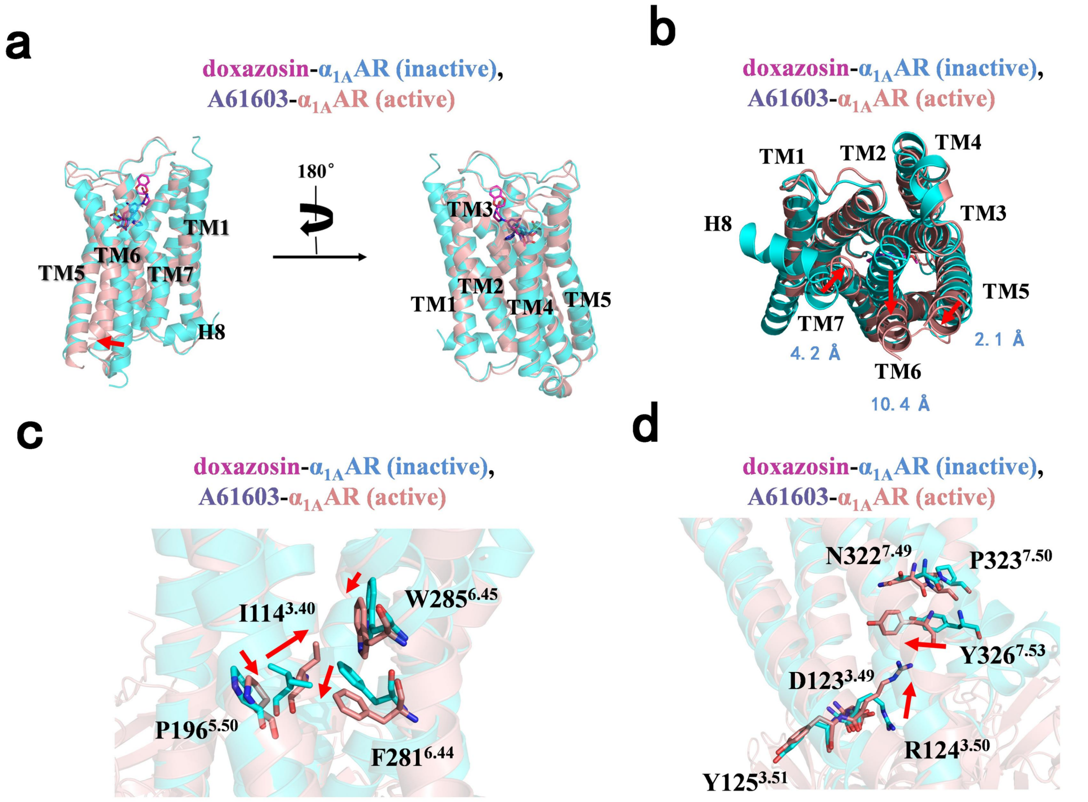


**Fig. S4: Conformational changes during $\alpha_{1A}$AR activation compared with the inactive state of $\alpha_{1A}$AR bound to doxazosin.**

**a.** Side view comparison of $\alpha_{1A}$AR bound to doxazosin and A61603 (blue-purple) (PDB ID:8THK). The $\alpha_{1A}$AR bound to A61603 is depicted in light pink. The red arrow illustrates the outward displacement of TM6 upon binding with A61603. **b.** Comparison of $\alpha_{1A}$AR bound to doxazosin and A61603 on the cytoplasmic face. The displacement distances of TM6, TM7, and TM5 were measured using the Cα atoms of residues A270$^{6.33}$, Y326$^{7.53}$, and R213$^{5.67}$, respectively. These are indicated in the figure with light-blue labels, and highlighted with red arrows. **c** Conformational changes of the toggle switch W285$^{6.48}$ and PIF motif. Residue shifts are indicated by red arrows. W285$^{6.48}$ (position of the 7th carbon on the indole ring) moves downward by 2.2 Å, P196$^{5.50}$ (position of Cα atom) shifts downward by 1.3 Å, and F281$^{6.44}$ (position of the 4th carbon on the benzene ring) moves outward by 5.3 Å. **d** Conformational changes of the NPxxY and DRY motifs. Residue shifts are marked by red arrows. R124$^{3.50}$ (position of the central carbon of the guanidinium group) shifts upward by 6.2 Å, and Y326$^{7.53}$ (position of Cα atom) moves inward toward the helix axis by 4.2 Å.

| | Ballesteros-Weinstein | | | | | | | | | | | | | | | | | | | | | | | | | | | |
|---|---|---|---|---|---|---|---|---|---|---|---|---|---|---|---|---|---|---|---|---|---|---|---|---|---|---|---|---|
| | 2.61 | 2.64 | 2.65 | 2.68 | 3.28 | 3.29 | 3.32 | 3.33 | 3.36 | 3.37 | 45.50 | 45.51 | 45.52 | 5.38 | 5.39 | 5.42 | 5.43 | 5.46 | 6.48 | 6.51 | 6.52 | 6.55 | 7.32 | 7.35 | 7.36 | 7.39 | 7.40 | 7.43 |
| **$\alpha_{1A}$AR** | S 83 | F 86 | E 87 | G 90 | W 102 | A 103 | D 106 | V 107 | C 110 | T 111 | C 176 | Q 177 | I 178 | Y 184 | V 185 | S 188 | A 189 | S 192 | W 285 | F 288 | F 289 | M 292 | E 305 | F 308 | K 309 | F 312 | W 313 | Y 316 |
| **$\alpha_{1B}$AR** | S | L | E | G | W | A | D | V | C | T | C | G | V | Y | A | S | S | S | W | F | F | L | D | F | K | F | W | Y |
| **$\alpha_{1D}$AR** | S | M | E | G | W | A | D | V | C | T | C | G | I | Y | A | S | S | S | W | F | F | L | E | F | K | F | W | Y |
| **$\alpha_{2A}$AR** | S | N | E | G | Y | L | D | V | C | T | C | E | I | Y | V | S | C | S | W | F | F | Y | R | F | K | F | W | Y |
| **$\alpha_{2B}$AR** | S | N | E | G | Y | L | D | V | C | T | C | K | L | Y | I | S | S | S | W | F | F | Y | H | F | Q | F | W | Y |
| **$\alpha_{2C}$AR** | S | N | E | A | Y | L | D | V | C | T | C | G | L | Y | I | S | C | S | W | F | F | Y | G | F | K | F | W | Y |

**Fig. S5: Comparison of amino acids in the ligand-binding pocket of $\alpha_{1A}$AR and related receptors.**

Comparison of the amino acid sequences in the ligand-binding pockets of $\alpha_{1A}$AR, $\alpha_{1B}$AR, $\alpha_{1D}$AR, $\alpha_{2A}$AR, $\alpha_{2B}$AR, and $\alpha_{2C}$AR. $\alpha_{1A}$AR: $\alpha_{1A}$-adrenoceptor, $\alpha_{1B}$AR: $\alpha_{1B}$-adrenoceptor, $\alpha_{1D}$AR: $\alpha_{1D}$-adrenoceptor, $\alpha_{2A}$AR: $\alpha_{2A}$-adrenoceptor, $\alpha_{2B}$AR: $\alpha_{2B}$-adrenoceptor, $\alpha_{2C}$AR: $\alpha_{2C}$-adrenoceptor.

a

| antagonist | $pK_i$ |
|---|---|
| silodosin | 9.6-10.4 |
| tamsulosin | 9.4-10.7 |
| doxazosin | 8.4-9.3 |

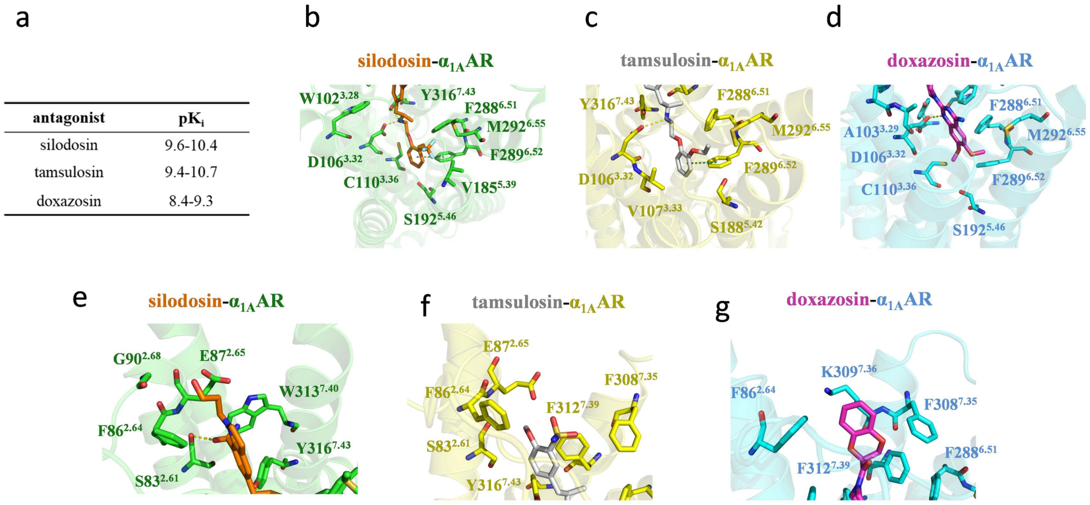


**Fig. S6: Affinity and partial interaction details of different antagonists with $\alpha_{1A}$AR.**

**a.** Affinity of silodosin, tamsulosin, and doxazosin for $\alpha_{1A}$AR (data obtained from https://www.guidetopharmacology.org/). Silodosin and tamsulosin generally exhibit higher affinity for $\alpha_{1A}$AR compared to doxazosin. **b-d.** Detailed interactions between $\alpha_{1A}$AR and the phenoxy-trifluoroethoxy moiety of silodosin (**b**), the ethoxy-phenoxy moiety of tamsulosin (**c**), and the dimethoxy-quinazolinyl moiety of doxazosin (**d**). Silodosin and tamsulosin form π-π interactions with F289$^{6.52}$ through deep insertion of their benzene rings into the binding pocket (**b-c**). The quinazoline moiety of doxazosin, possessing a planar rigid structure, cannot insert effectively into the binding pocket, precluding a similar π–π interaction with residue F289$^{6.52}$ (**d**). **e-g.** Detailed interactions between $\alpha_{1A}$AR and silodosin's hydroxypropyl indole carboxamide (**e**), tamsulosin's methoxybenzenesulfonamide (**f**), and doxazosin's 1,4-benzodioxan-2-carbonyl moieties (**g**). Van der Waals interactions mediated by residue E87$^{2.65}$ of $\alpha_{1A}$AR were observed exclusively in the complexes with silodosin and tamsulosin (**e-f**), but not with doxazosin (**g**). Hydrogen bonding with residue S83$^{2.61}$ was uniquely detected in the silodosin complex (**e**). **b-g.** Hydrogen bonds are represented as yellow dash lines, while π–π interactions are depicted as deep green lines.

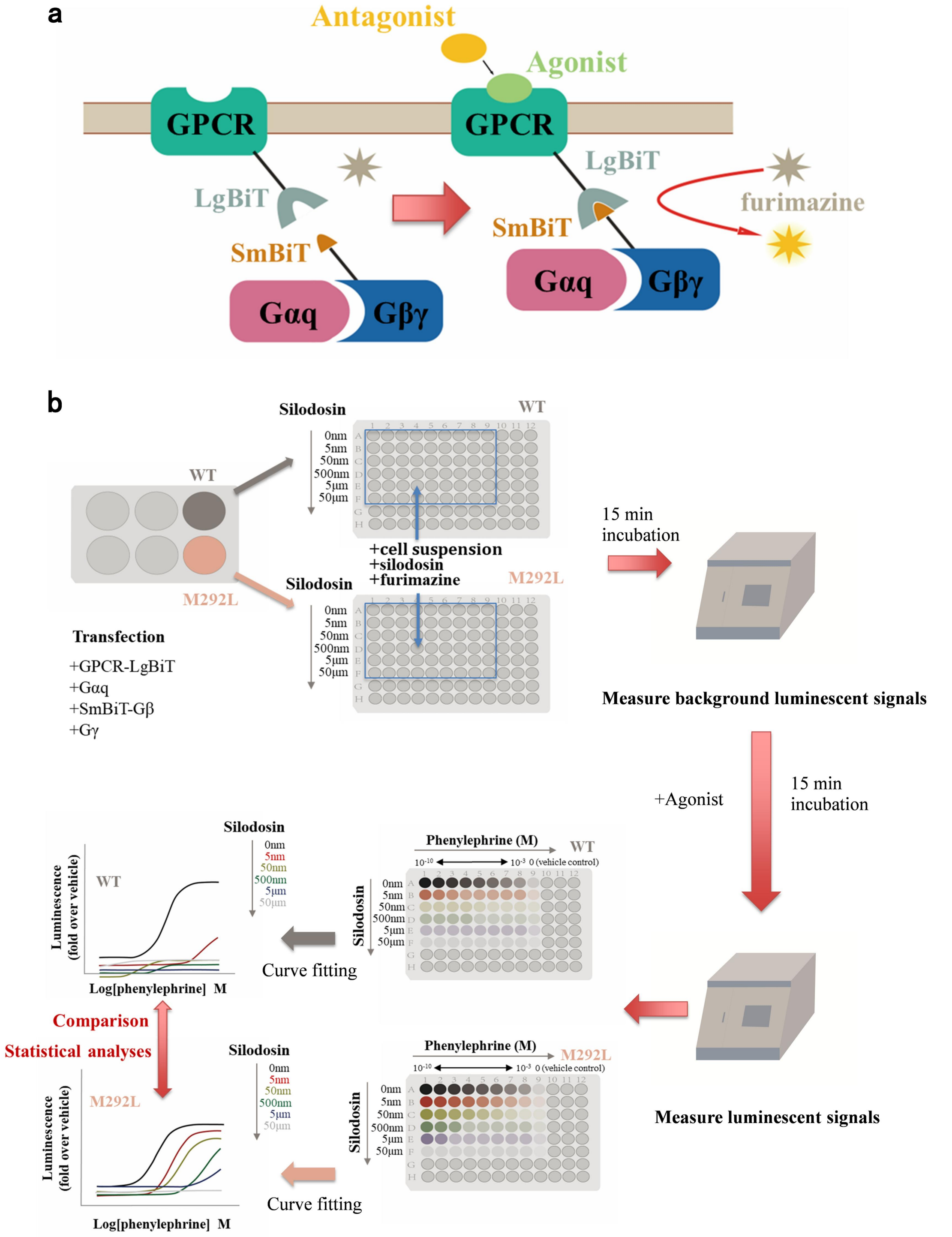


**Fig. S7: Principle, experimental design, and workflow of NanoBiT recruitment experiments.**

**a.** Principle of NanoBiT recruitment experiments. **b.** Experimental design and workflow of NanoBiT recruitment experiments, illustrated using wild-type $\alpha_{1A}$AR, the $\alpha_{1A}$AR mutant M292L, and the antagonist silodosin as representative examples.

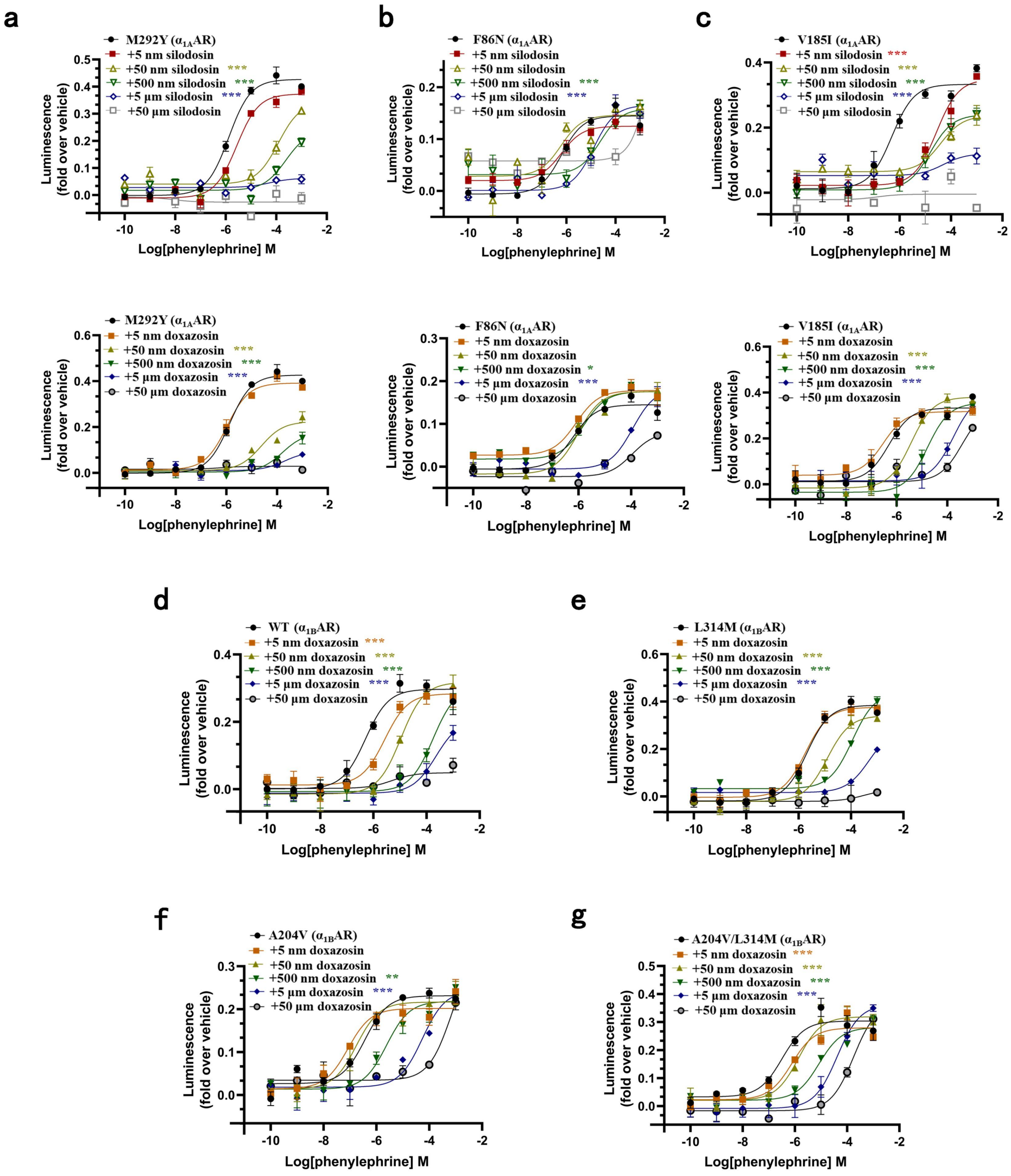


**Fig. S8: Functional assays of mutations for silodosin subtype selectivity.**
**a-c.** The inhibitory effects of varying concentrations of silodosin and doxazosin on $\alpha_{1A}$AR mutants M292Y, F86N, and V185I, which were assessed through NanoBiT recruitment experiments. **d-g.** The inhibitory effects of different concentrations of doxazosin on wild-type $\alpha_{1B}$AR and $\alpha_{1B}$AR mutants L314M, A204V, and A204V/L314M, which were evaluated through NanoBiT recruitment experiments. **a-g.** Luminescence data (fold over vehicle), averaged from NanoBiT recruitment assays (n=3), were used as input for plotting. Agonist activation curves for phenylephrine were then fitted using a three-parameter logistic equation. Activation curves are shown for various concentrations of antagonist, differentiated by color and shape. Activation curves in the absence of antagonist are indicated in black. Data are presented as mean ± SD. Statistically significant differences between activation curves in the presence and absence of a specific

concentration of antagonist were determined by comparing the -LogEC50 values obtained from three separately fitted curves using one-way analysis of variance (ANOVA), followed by Dunnett's multiple comparison test. (*P < 0.05, **P < 0.01, ***P < 0.001). Due to inadequate activation of several experimental groups at an antagonist concentration of 50 μM, these data were omitted from subsequent statistical analysis to maintain uniformity.

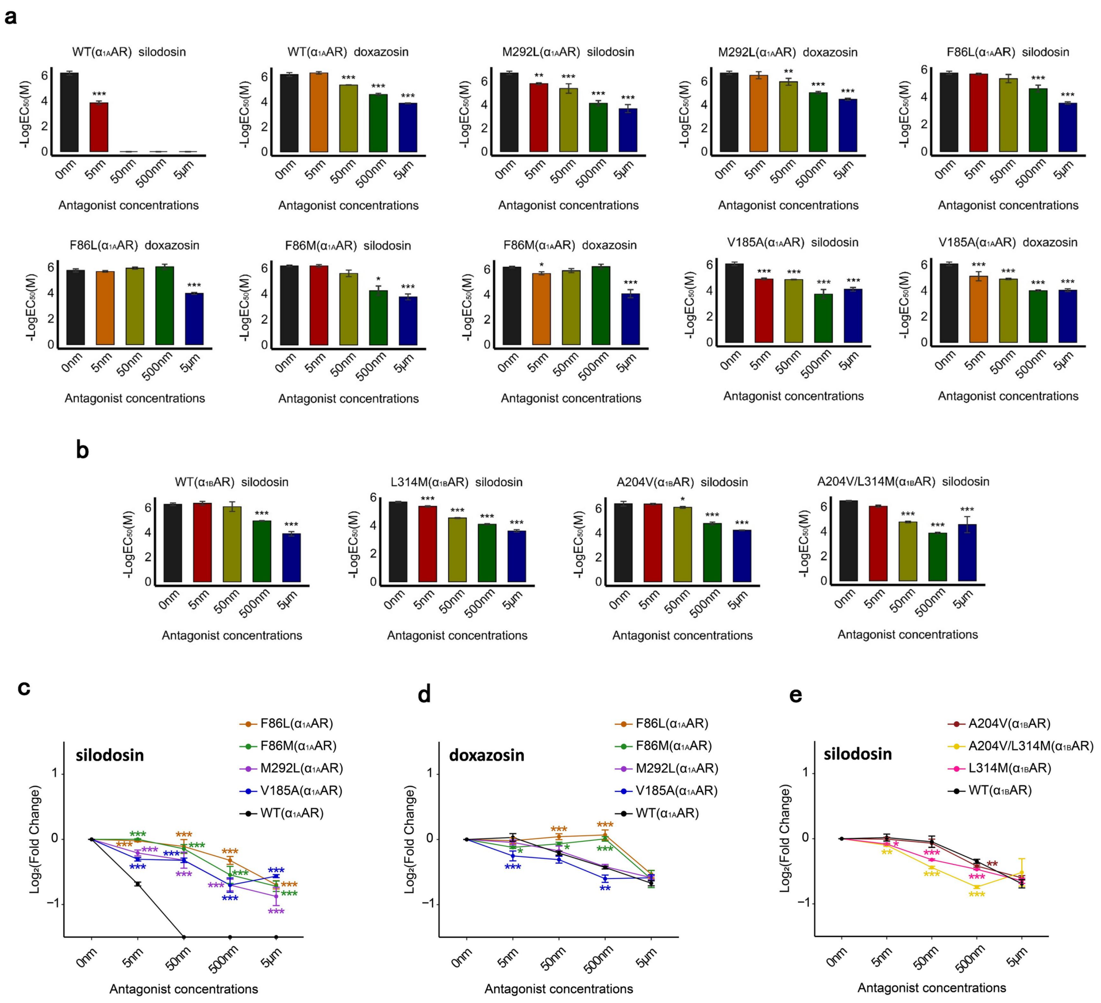


**Fig. S9: Functional validation of silodosin subtype selectivity through mutational analysis. (statistical analysis).**

**a-b.** Comparison of $-LogEC_{50}$ values at varying antagonist concentrations. -LogEC50 values, derived from three separately fitted curves, were used to generate the bar graph, with different colors representing different antagonist concentrations. Data are presented as mean ± SD. Asterisks denote statistically significant differences in $-LogEC_{50}$ compared to the condition without antagonist (0 nM) (one-way analysis of variance (ANOVA), followed by Dunnett's multiple comparison test, *P < 0.05, **P < 0.01, * **P < 0.001, displayed for experimental groups where the mean difference in $-LogEC_{50}$ relative to the 0 nM condition was less than 0). In the "WT($\alpha_{1A}$AR) silodosin" bar graph, activation curves at antagonist concentrations of 50 nM and above were classified as non-activatable because the luminescence (fold over vehicle) remained below 0, even at the maximum agonist concentration. Consequently, the $-LogEC_{50}$ for these curves was defined as the lower boundary of the coordinate axis. **c-e.** The fold change in $-LogEC_{50}$ values was compared across different receptor subtypes as the antagonist concentration increased. Trends in $EC_{50}$ variation were assessed by calculating the $log_2$(Fold Change), which represents the logarithm of the fold change in $-LogEC_{50}$ at various antagonist concentrations relative to the $-LogEC_{50}$

in the absence of antagonist (0 nM). Data were obtained from three separately fitted curves and are presented as mean ± SD. Different colors represent different mutants. Asterisks indicate statistically significant differences between mutants and the wild-type (WT) at the same antagonist concentration (one-way analysis of variance (ANOVA), followed by Dunnett's multiple comparison test, $*P < 0.05$, $**P < 0.01$, $***P < 0.001$). The $\log_2$(fold change) for inactive curves in (**c**) was defined as the lower boundary of the coordinate axis.

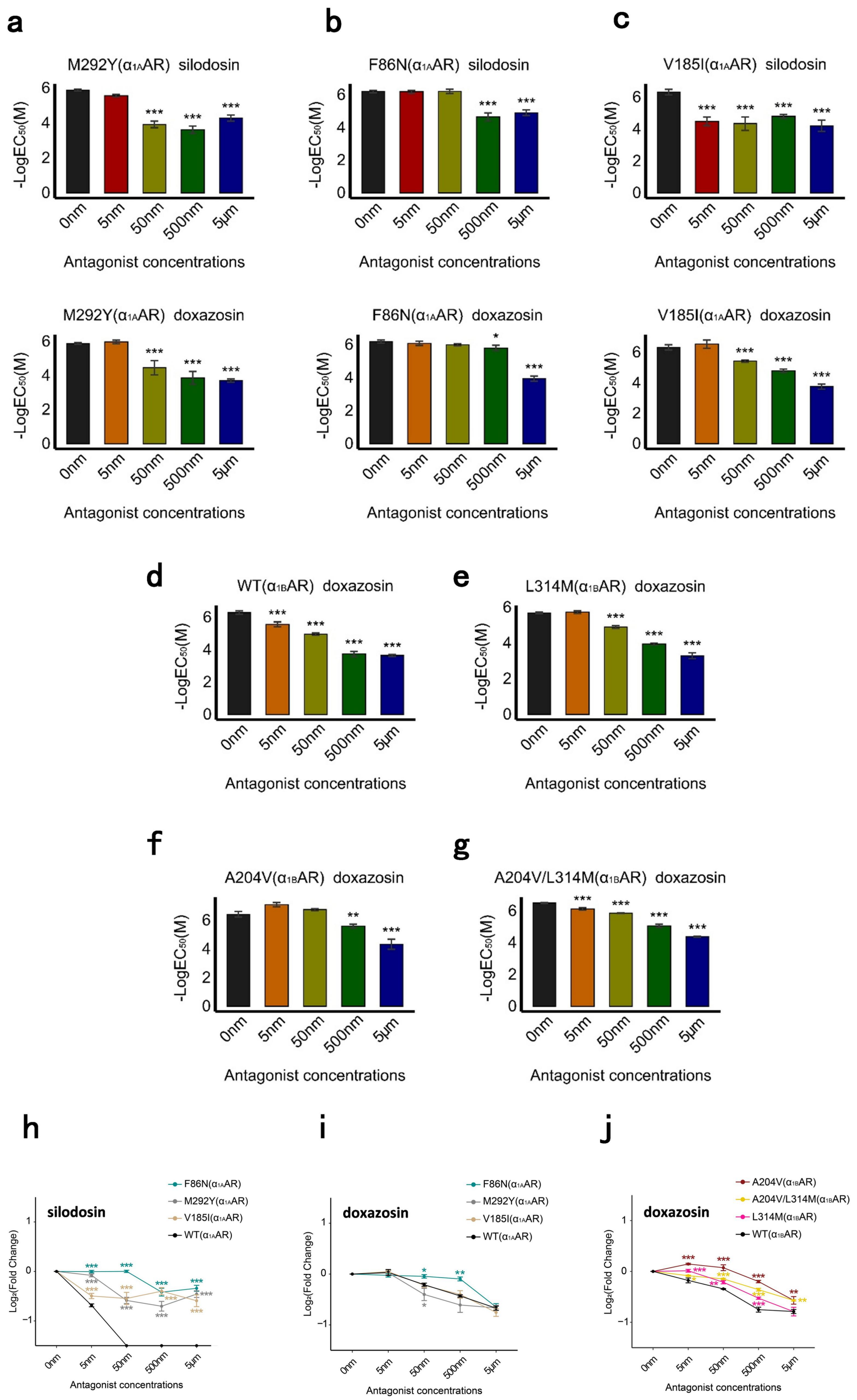


**Fig. S10: Functional assays of mutations for silodosin subtype selectivity (statistical analysis).**

**a-g.** Comparison of $-LogEC_{50}$ values at varying antagonist concentrations. -

LogEC$_{50}$ values, derived from three separately fitted curves, were used to generate the bar graph, with different colors representing different antagonist concentrations. Data are presented as mean ± SD. Asterisks denote statistically significant differences in -LogEC$_{50}$ compared to the condition without antagonist (0 nM) (one-way analysis of variance (ANOVA), followed by Dunnett's multiple comparison test, *P < 0.05, **P < 0.01, * **P < 0.001, displayed for experimental groups where the mean difference in -LogEC$_{50}$ relative to the 0 nM condition was less than 0). **h-j.** The fold change in -LogEC$_{50}$ values was compared across different receptor subtypes as the antagonist concentration increased. Trends in EC$_{50}$ variation were assessed by calculating the log$_2$(Fold Change), which represents the logarithm of the fold change in -LogEC$_{50}$ at various antagonist concentrations relative to the -LogEC$_{50}$ in the absence of antagonist (0 nM). Data were obtained from three separately fitted curves and are presented as mean ± SD. Different colors represent different mutants. Asterisks indicate statistically significant differences between mutants and the wild-type (WT) at the same antagonist concentration (one-way analysis of variance (ANOVA), followed by Dunnett's multiple comparison test, *P < 0.05, **P < 0.01, ***P < 0.001). The log$_2$(fold change) for inactive curves in (**h**) was defined as the lower boundary of the coordinate axis.

**Table S1**: **Cryo-EM data collection, model refinement and validation statistics.**

| Data collection/processing | Doxazosin -$\alpha_{1A}$AR-mBRIL | Silodosin -$\alpha_{1A}$AR-mBRIL |
|---|---|---|
| Magnification | 81,000 | 81,000 |
| Voltage (kV) | 300 | 300 |
| Electron exposure ($e^-$/Å$^2$) | 50 | 50 |
| Defocus range (μm) | -1.7~-2.3 | -1.7~-2.3 |
| Pixel size (Å) | 0.413 | 0.413 |
| Symmetry imposed | C1 | C1 |
| Initial particle projections (no.) | 3,997,625 | 3,759,439 |
| Final particle projections (no.) | 132,158 | 132,548 |
| Map resolution (Å) | 2.99 | 3.19 |
| FSC threshold | 0.143 | 0.143 |
| **Refinement** | | |
| Initial model used | Alphafold2 | Alphafold2 |
| Model resolution (Å) | 3.5 | 3.8 |
| FSC threshold | 0.5 | 0.5 |
| Model resolution range (Å) | 50-3.1 | 50-3.1 |
| Map sharpening B factor (Å$^2$) | -104.1 | -114.7 |
| **Model composition** | | |
| Non-hydrogen atoms | 2210 | 2210 |
| Protein residues | 277 | 277 |
| Ligand | 1 | 1 |
| **B factors (Å$^2$)** | | |
| Protein | 28.70 | 91.62 |
| Ligand | 34.22 | 91.39 |
| **R.m.s. deviations** | | |
| Bond lengths (Å) | 0.003 | 0.003 |
| Bond angles (°) | 0.562 | 0.616 |
| **Validation** | | |
| MolProbity score | 1.69 | 1.58 |
| Clashs core | 11.62 | 11.57 |
| Rotamer outliers (%) | 0.00 | 0.00 |
| **Ramachandran plot** | | |
| Favored (%) | 97.44 | 98.92 |
| Allowed (%) | 2.56 | 1.08 |

**Movie S1:** Pymol morph video illustrating the conformational changes of $\alpha_{1A}$AR transitioning from the silodosin-bound inactive state to the A61603-bound active state. $\alpha_{1A}$AR is shown in green, and A61603 is depicted in blue-purple.
**Movie S2:** Pymol morph video illustrating the conformational changes of $\alpha_{1A}$AR transitioning from the doxazosin-bound inactive state to the A61603-bound active state. $\alpha_{1A}$AR is displayed in light blue, and A61603 is displayed in blue-purple.

**Data S1:** Key metrics of activation curves (-Log$EC_{50}$, fold change and related data).
**Data S2:** Bar plots statistical analysis results.
**Data S3:** Line plots statistical analysis results.
**Data S4:** The amino acid sequences of the recombinant expression vectors used in this study.
