# Supplementary material for: Molecular mechanism of antagonist recognition and regulation of the α1A-adrenoceptor
Source: J Biol Chem. 2025 Jun 6;301(7):110348. doi: 10.1016/j.jbc.2025.110348 (PMC12269490; doi:10.1016/j.jbc.2025.110348)
Supplement: Supplementary Data 4 [file mmc5.docx]

**The amino acid sequences of the** **recombinant expression vectors used in this study**

Fab

MKYLLPTAAAGLLLLAAQPAMADIQMTQSPSSLSASVGDRVTITCRASQSVSSAVAWYQQKPGKAPKLLIYSASSLYSGVPSRFSGSRSGTDFTLTISSLQPEDFATYYCQQPWSWGSALITFGQGTKVEIKRTVAAPSVFIFPPSDSQLKSGTASVVCLLNNFYPREAKVQWKVDNALQSGNSQESVTEQDSKDSTYSLSSTLTLSKADYEKHKVYACEVTHQGLSSPVTKSFNRGEVQLVESGGGLVQPGGSLRLSCAASGFNIYYSSMHWVRQAPGKGLEWVASISPYSGSTYYADSVKGRFTISADTSKNTAYLQMNSLRAEDTAVYYCARPWYPWSYWSGLDYWGQGTLVTVSSASTKGPSVFPLAPSSKSTSGGTAALGCLVKDYFPEPVTVSWNSGALTSGVHTFPAVLQSSGLYSLSSVVTVPSSSLGTQTYICNVNHKPSNTKVDKKVEPKSHHHHHH

Black: PelB Signal Peptide; Red: Light Chain; Blue: Heavy Chain; Purple: His-tag

G7-13

MKYLLPTAAAGLLLLAAQPAMAEIAALEKEIAALEKEIAALEKGSGSGSGQVQLQESGGGLVQPGGSLRLSCAASGRTISRYAMSWFRQAPGKEREFVAVARRSGDGAFYADSVQGRFTVSRDDAKNTVYLQMNSLKPEDTAVYYCAIDSDTFYSGSYDYWGQGTQVTVSSGSGSGSSGSGSSGEVQLQESGGGLVQPGGSLRLSCTASGVTISALNAMAMGWYRQAPGERRVMVAAVSERGNAMYRESVQGRFTVTRDFTNKMVSLQMDNLKPEDTAVYYCHVLEDRVDSFHDYWGQGTQVTVSSHHHHHH

Black: PelB Signal Peptide; Red: E3 coil; Orange: 7xGS linker; Blue: NbFab; Green: 13xGS linker; Pink: ALFANb; Purple: His-tag

**α_1A_AR-mBRIL**

MKTIIALSYIFCLVFADYKDDDDKSGNASDSSNCTQPPAPVNISKAILLGVILGGLILFGVLGNILVILSVACHRHLHSVTHYYIVNLAVADLLLTSTVLPFSAIFEVLGYWAFGRVFCNIWAAVDVLCCTASIMGLCIISIDRYIGVSYPLRYPTIVTQRRGLMALLCVWALSLVISIGPLFGWRQPAPEDETICQINEEPGYVLFSALGSFYLPLAIILVMYCRVYVVAKRELADLEDNWETLNDNLKVIEKADNAAQVKDALTKMRAAALDAQKASGSGSPEMKDFRHGFDILVGQIDDALKLANEGKVKEAQAAAEQLKTTRNAYIQKYLLKFSREKKAAKTLGIVVGCFVLCWLPFFLVMPIGSFFPDFKPSETVFKIVFWLGYLNSCINPIIYPCSSQEFKKAFQNVLKIAALKEKIAALKEKIAALKEAEEKRASRLEEELRRRLTEHHHHHHHH

Green: HA Signal Peptide; Orange: Flag Tag; Red: α_1A_AR; Blue: mBRIL; Purple: His-tag

**α_1A_AR -LgBiT**

MKTIIALSYIFCLVFASGNASDSSNCTQPPAPVNISKAILLGVILGGLILFGVLGNILVILSVACHRHLHSVTHYYIVNLAVADLLLTSTVLPFSAIFEVLGYWAFGRVFCNIWAAVDVLCCTASIMGLCIISIDRYIGVSYPLRYPTIVTQRRGLMALLCVWALSLVISIGPLFGWRQPAPEDETICQINEEPGYVLFSALGSFYLPLAIILVMYCRVYVVAKRESRGLKSGLKTDKSDSEQVTLRIHRKNAPAGGSGMASAKTKTHFSVRLLKFSREKKAAKTLGIVVGCFVLCWLPFFLVMPIGSFFPDFKPSETVFKIVFWLGYLNSCINPIIYPCSSQEFKKAFQNVLGGSGGGGSGGSSSGGVFTLEDFVGDWEQTAAYNLDQVLEQGGVSSLLQNLAVSVTPIQRIVRSGENALKIDIHVIIPYEGLSADQMAQIEEVFKVVYPVDDHHFKVILPYGTLVIDGVTPNMLNYFGRPYEGIAVFDGKKITVTGTLWNGNKIIDERLITPDGSMLFRVTINS

Green: HA Signal Peptide; Red: α_1A_AR; Green: 15xGS linker; Blue: LgBiT

**α_1B_AR -LgBiT**

MKTIIALSYIFCLVFANANFTGPNQTSSNSTLPQLDITRAISVGLVLGAFILFAIVGNILVILSVACNRHLRTPTNYFIVNLAMADLLLSFTVLPFSAALEVLGYWVLGRIFCDIWAAVDVLCCTASILSLCAISIDRYIGVRYSLQYPTLVTRRKAILALLSVWVLSTVISIGPLLGWKEPAPNDDKECGVTEEPFYALFSSLGSFYIPLAVILVMYCRVYIVAKRTTKNLEAGVMKEMSNSKELTLRIHSKNFHEDTLSSTKAKGHNPRSSIAVKLFKFSREKKAAKTLGIVVGMFILCWLPFFIALPLGSLFSTLKPPDAVFKVVFWLGYFNSCLNPIIYPCSSKEFKRAFVRILGCQCGGSGGGGSGGSSSGGVFTLEDFVGDWEQTAAYNLDQVLEQGGVSSLLQNLAVSVTPIQRIVRSGENALKIDIHVIIPYEGLSADQMAQIEEVFKVVYPVDDHHFKVILPYGTLVIDGVTPNMLNYFGRPYEGIAVFDGKKITVTGTLWNGNKIIDERLITPDGSMLFRVTINS

Green: HA Signal Peptide; Red: α_1B_AR; Green: 15xGS linker; Blue: LgBiT

**Gαq**

MTLESIMACCLSEEAKEARRINDEIERQLRRDKRDARRELKLLLLGTGESGKSTFIKQMRIIHGSGYSDEDKRGFTKLVYQNIFTAMQAMIRAMDTLKIPYKYEHNKAHAQLVREVDVEKVSAFENPYVDAIKSLWNDPGIQECYDRRREYQLSDSTKYYLNDLDRVADPAYLPTQQDVLRVRVPTTGIIEYPFDLQSVIFRMVDVGGQRSERRKWIHCFENVTSIMFLVALSEYDQVLVESDNENRMEESKALFRTIITYPWFQNSSVILFLNKKDLLEEKIMYSHLVDYFPEYDGPQRDAQAAREFILKMFVDLNPDSDKIIYSHFTCATDTENIRFVFAAVKDTILQLNLKEYNLV

Black: Gq

**SmBiT-Gβ**

MVTGYRLFEEILGGSGGGGSGGSSSGGSELDQLRQEAEQLKNQIRDARKACADATLSQITNNIDPVGRIQMRTRRTLRGHLAKIYAMHWGTDSRLLVSASQDGKLIIWDSYTTNKVHAIPLRSSWVMTCAYAPSGNYVACGGLDNICSIYNLKTREGNVRVSRELAGHTGYLSCCRFLDDNQIVTSSGDTTCALWDIETGQQTTTFTGHTGDVMSLSLAPDTRLFVSGACDASAKLWDVREGMCRQTFTGHESDINAICFFPNGNAFATGSDDATCRLFDLRADQELMTYSHDNIICGITSVSFSKSGRLLLAGYDDFNCNVWDALKADRAGVLAGHDNRVSCLGVTDDGMAVATGSWDSFLKIWN

Red: SmBiT; Green: 15xGS linker; Blue: Gβ

**Gγ**

MASNNTASIAQARKLVEQLKMEANIDRIKVSKAAADLMAYCEAHAKEDPLLTPVPASENPFREKKFFCAIL

Black: Gγ
